# *Iris lactea* var. *chinensis* plant drought tolerance depends on the response of proline metabolism, transcription factors, transporters and the ROS-scavenging system

**DOI:** 10.1186/s12870-022-04019-4

**Published:** 2023-01-09

**Authors:** Yue Zhang, Ruihai Zhang, Zhen Song, Weidong Fu, Lingling Yun, Jinhui Gao, Guang Hu, Zhonghui Wang, Hanwen Wu, Guoliang Zhang, Jiahe Wu

**Affiliations:** 1 Institute of Environment and Sustainable Development in Agriculture, Chinese Academy of Agricultural Sciences, Beijing, 100081 China; 2 State Key Laboratory of Plant Genomics, Institute of Microbiology, Chinese Academy of Sciences, Beijing, 100101 China; 3 Wagga Wagga Agricultural Institute, NSW Department of Primary Industries, Wagga Wagga, New South Wales 2650 Australia

**Keywords:** *Iris lactea* var. *chinensis*, Drought stress, Rehydration, Transcriptome, Transcription factor

## Abstract

**Background:**

*Iris lactea* var. *chinensis*, a perennial herbaceous species, is widely distributed and has good drought tolerance traits. However, there is little information in public databases concerning this herb, so it is difficult to understand the mechanism underlying its drought tolerance.

**Results:**

In this study, we used Illumina sequencing technology to conduct an RNA sequencing (RNA-seq) analysis of *I. lactea* var. *chinensis* plants under water-stressed conditions and rehydration to explore the potential mechanisms involved in plant drought tolerance. The resulting de novo assembled transcriptome revealed 126,979 unigenes, of which 44,247 were successfully annotated. Among these, 1187 differentially expressed genes (DEGs) were identified from a comparison of the water-stressed treatment and the control (CK) treatment (T/CK); there were 481 upregulated genes and 706 downregulated genes. Additionally, 275 DEGs were identified in the comparison of the rehydration treatment and the water-stressed treatment (R/T). Based on Quantitative Real-time Polymerase Chain Reaction (qRT-PCR) analysis, the expression levels of eight randomly selected unigenes were consistent with the transcriptomic data under water-stressed and rehydration treatment, as well as in the CK. According to Gene Ontology (GO) annotation and Kyoto Encyclopedia of Genes and Genomes (KEGG) pathway analysis, proline metabolism-related DEGs, including those involved in the ‘proline catabolic process’, the ‘proline metabolic process’, and ‘arginine and proline metabolism’, may play important roles in plant drought tolerance. Additionally, these DEGs encoded 43 transcription factors (TFs), 46 transporters, and 22 reactive oxygen species (ROS)-scavenging system-related proteins. Biochemical analysis and histochemical detection showed that proline and ROS were accumulated under water-stressed conditions, which is consistent with the result of the transcriptomic analysis.

**Conclusions:**

In summary, our transcriptomic data revealed that the drought tolerance of *I. lactea* var. *chinensis* depends on proline metabolism, the action of TFs and transporters, and a strong ROS-scavenging system. The related genes found in this study could help us understand the mechanisms underlying the drought tolerance of *I. lactea* var. *chinensis*.

**Supplementary Information:**

The online version contains supplementary material available at 10.1186/s12870-022-04019-4.

## Background

Drought is one of the most severe and frequently occurring abiotic stresses threatening plant seed germination, growth, and productivity. However, throughout the long-term course of the evolution, plants have developed complex and sophisticated systems to counteract drought stress, including various physiological, biochemical, metabolic, and tolerance mechanisms that are enabled through changes in the expression patterns of thousands of genes [1–5].

The genes involved in mechanisms of drought stress adaptation can be grouped according to their functions with respect to three major strategies [6]. The first strategy consists of genes concerned with the direct protection of essential proteins and membranes, as performed by osmoprotectants, free radical scavengers, and heat shock proteins. Under drought stress, the accumulation of reactive oxygen species (ROS), including singlet oxygen (^1^O_2_), superoxide (O_2_^−^), hydroxyl (−OH), and hydrogen peroxide (H_2_O_2_), can cause oxidative stress in plants, which induces the degradation of pigments, proteins, lipids and nucleic acids, resulting in cell death [7, 8]. Therefore, plants are subjected to damage by oxidative drought-induced stress, as described by Mano [9]. To avert excess ROS, plant cells are equipped with antioxidative machinery composed of both enzymes and low molecular weight compounds. The antioxidant enzymes, including superoxide dismutase (SOD), catalase (CAT), ascorbate peroxidase (APX), play crucial roles in maintaining ROS homeostasis [10, 11]. The second strategy comprises the increase activity of membrane transporters and ion channels, that are involved in water and ion uptake. Membrane transporters were found to be the important modulators in the translocation of ABA and osmotic adjustment associated with drought stress [12]. The overexpression of AtABCG25/WBC26 transporter, an ABA Efflux carrier, can reduce water loss from leaves by facilitating the delivery of ABA to guard cells [13]. The AtABCG40 transporter, which imports ABA into stomatal cells, has been proven to maintain the sensitivity of guard cells to ABA under drought stress [14]. On the other hand, most plants could tolerate drought stress by increasing cellular osmolarity, which regulates by inorganic ions and compatible solutes (sugars, sugar alcohols, amino acids, and quaternary ammonium, etc.) [15]. Membrane transporters are associated with the regulation of movement and differential distribution of these compounds [12]. The third strategy consists of regulatory proteins, including kinases and transcription factors (TFs) that are involved in the transcriptional regulation of stress-related genes. Plant TFs play an important role in plant drought tolerance, directly regulating the gene expression and serving as molecular switches [16], involving in a wide range of TF family members, such as WRKY, MYB, NAC, bZIP, and bHLH [17]. Increasing evidence indicates that many transporters act as major players in plant drought tolerance; e.g., various plant membrane transport systems (those involving ABC transporters, sugar transporters, and so forth) play a substantial role in adaptation to drought [14, 18]. For example, sulfate transporters (SULTRs) may contribute to adjusting the sulfur distribution in plants subjected to drought stress [19].

*Iris lactea* var. *chinensis*, a perennial herbaceous species, is widely distributed in northern China, regions of Siberian, eastern Russia, and Mongolia [20]. This plant grows on desert steppes, in wastelands, in parks; this species has strong adaptability to the environment and has been used to restore degraded fields and landscaping plant [21]. *I. lactea* var. *chinensis* is more drought tolerant than are other plants species used for roadside ecological restoration, such as *Parthenocissus quinquefolia* and *Opisthopappus taihangensis*, according to evaluations of comprehensive physiological indexes under various degrees of stress [22]. To date, most research on *I. lactea* has focused on the screening of germplasm resources and the mechanisms underlying their salt-, heavy metal- and polyethylene glycol (PEG)-mediated drought tolerance [23–26]. However, the effects of simulated by PEG-mediated drought stress and direct drought stress (severe dehydration or withholding of water) are likely not be the same, considering the differences in the plant transcriptomes [27]. Moreover, some reports concerning the response of *I. lactea* var. *chinensis* to drought stress have focused only on morphological characteristics and basic physiological indicators, such as protective enzyme activity and the chlorophyll content [22, 28]. Therefore, there is a critical need to explore the underlying molecular mechanisms of *I. lactea* var. *chinensis* under water-stressed (severe dehydration or withholding of water).

In this study, we comprehensively characterized the mechanisms underlying the drought tolerance of *I. lactea* var. *chinensis*, and we analyzed the transcriptomes of leaf samples from plants under water-stressed (T), rehydrated plants (R), and the control (CK) plants. The resulting RNA sequencing (RNA-seq) data revealed that the drought tolerance of *I. lactea* var. *chinensis* depends on proline metabolism, the action of TFs and transporters, and a strong ROS-scavenging system, which may provide theoretical information concerning insights on the mechanisms underlying the drought tolerance of *I. lactea* var. *chinensis*.

## Results

### Phenotypic characterization

**Fig. 1:**
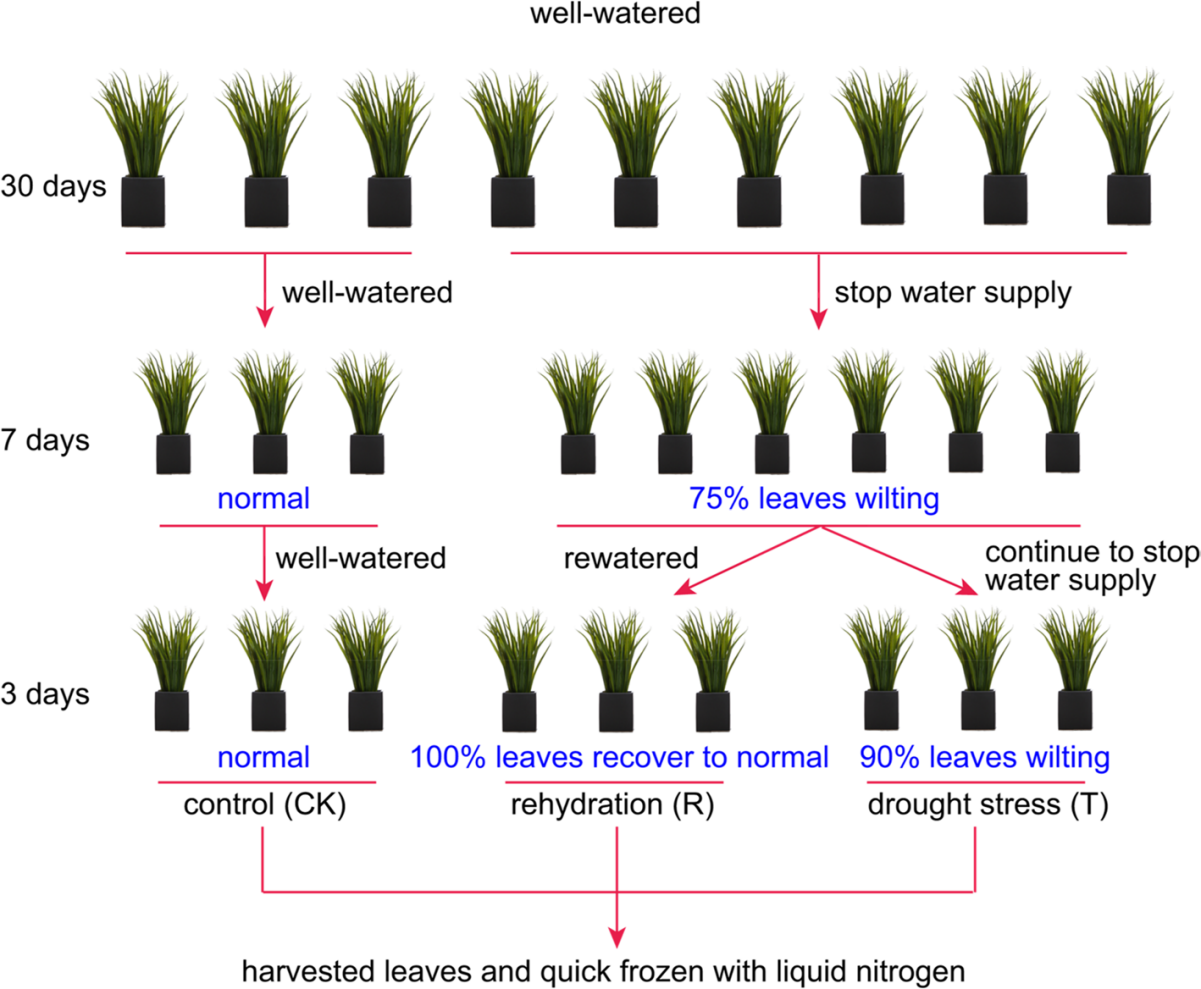
A scheme representing the experimental design

**Fig. 2:**
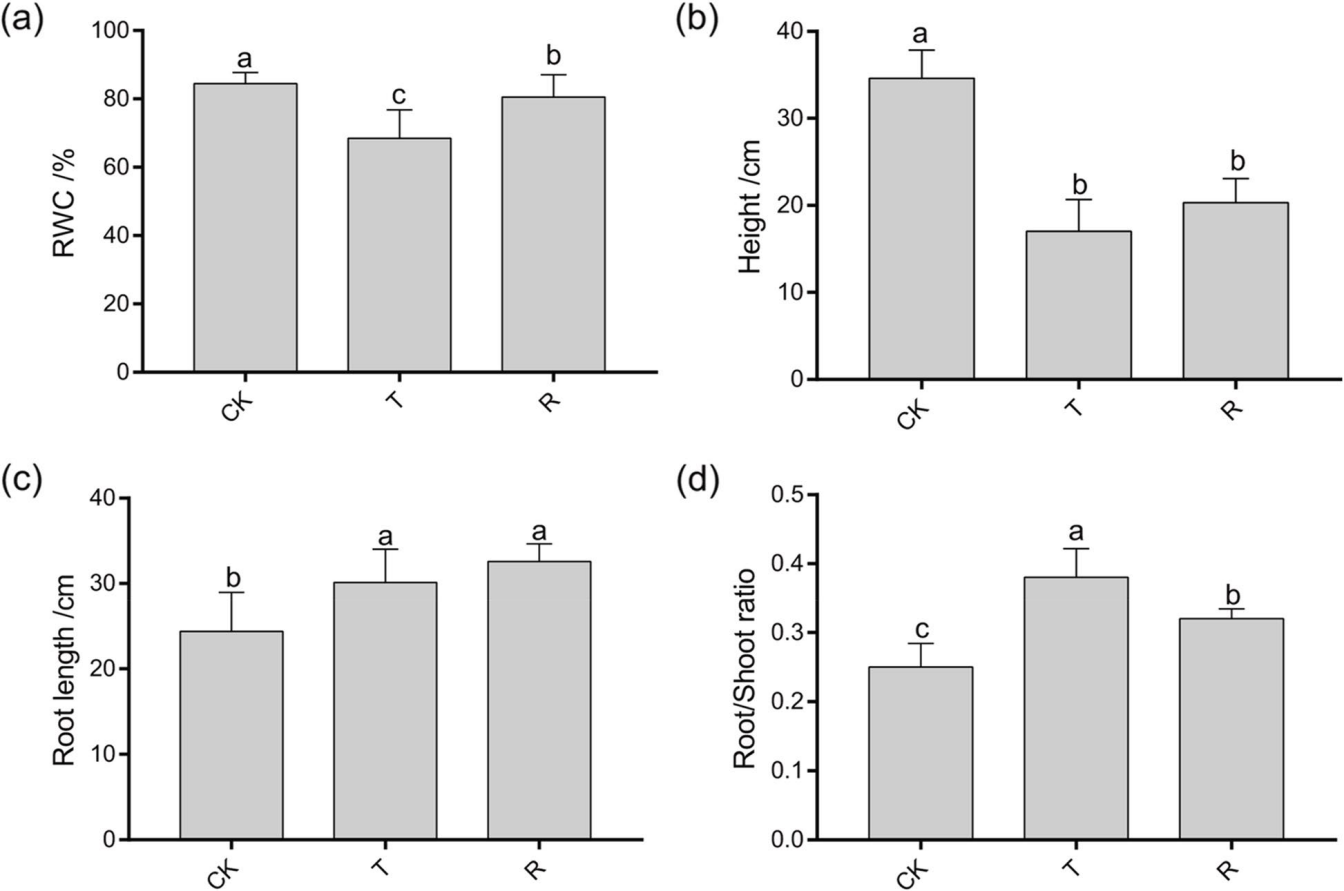
The phenotypic characterization (**a**) relative water content (RWC) of aerial parts, (**b**) plant height, (**c**) root length, and (**d**) root/shoot ratio of *Iris lactea* var. *chinensis* under normal watering (CK), water-stressed (T), and rehydration -treated (R) condition

*I. lactea* var. *chinensis* is a drought tolerant plant species that and can grow on desert steppes in and saline lowland meadows. To validate its drought tolerance, we performed a native water-stressed and rehydration experiment. The results showed that approximately 75% *I. lactea* var. *chinensis* plants were wilted after 7 days of natural water-stressed, and when they were rehydrated, all the wilted plants recovered after 3 days (Fig. 1). The relative water content (RWC) of the water-stressed plants significantly decreased by 18.92% compared to that of the CK plants (*p* < 0.05) (Fig. 2a). Additionally, the height of water-stressed plants was significantly lower (by 50.82%) than that of the CK plants (*p* < 0.05) (Fig. 2b). When the water-stressed plants were rehydrated, the RWC significantly increased by 17.58% compared to that of the drought-stress plants (p < 0.05) and recovered to 95.34% of the CK treatment level (Fig. 2a). In addition, the plant height in part increased by 17.58%; the plant height did not differ from that of the drought-stress treatment and was 41.29% lower than that under the drought-stress treatment (*p* < 0.05) (Fig. 2b). To further investigate the phenotypes of plants treated and not subjected to water-stressed, we measured the root length, root height, and shoot height, respectively. Compared to the CK plants (*p* < 0.05) (Fig. 2d), the root length and root/shoot ratio of the water-stressed plants significantly increased by 23.51% (Fig. 2c) and 52.00%, respectively, which were similar to those of other plants under water-stressed [29]. When the water-stressed plants were rehydrated, their root length slightly increased (by 8.21%) (Fig. 2c). In addition, the root/shoot ratio significantly decreased by 15.79% compared to that of plants under drought-stress treatment (p < 0.05); however, the ratio was still significantly higher (by 28.00%) than that of plants under the CK treatment (p < 0.05) (Fig. 2d).

### Primary transcriptome analysis

Three sample groups, the control (CK, normal watering), water-stressed (T) and rehydration-treated (R) with three biological replicates each were used to perform RNA-sequencing analysis using an Illumina HiSeq platform. A total of 57.85 Gb of data was obtained, and the CK, T, and R sample groups contained 42,979,607, 42,693,515, and 42,884,268 raw reads, respectively. Then, 42,905,695, 42,668,270, and 42,857,576 clean reads were obtained in the CK, T, and R groups, respectively, after filtering and removing the low-quality reads. Following this, de novo transcriptome assembly with Trinity revealed 126,979 unigenes, with an N50 of 1176 bp. These unigenes had an average length of 810 bp and an average GC content of 42.55% (Additional file 1: Table S1), indicating that they were of fine quality and suitable for further annotations. Annotations of the assembled unigenes were carried out according to information within six public databases, namely, the nonredundant (NR), SwissProt, Kyoto Encyclopedia of Genes and Genomes (KEGG), Clusters of Orthologous Groups of proteins (COG), Pfam, and Gene Ontology (GO) databases; in total, 41,360, 31,799, 18,512, 8875, 28,920 and 28,385 unigenes were aligned with the sequence information of these databases, respectively (Table 1). Finally, 44,247 unigenes (34.85%) were successfully annotated across the six databases.

**Table 1:** Number of functional annotations for all unigenes

Values	Total	NR	SwissProt	KEGG	COG	Pfam	GO	Overall
Number	126,979	41,360	31,799	18,512	8875	28,920	28,385	44,247
Percentage	100%	32.57%	25.04%	14.58%	6.99%	22.78%	22.35%	34.85%

### Analysis of the validity of the transcriptomic data by qRT-PCR

**Fig. 3:**
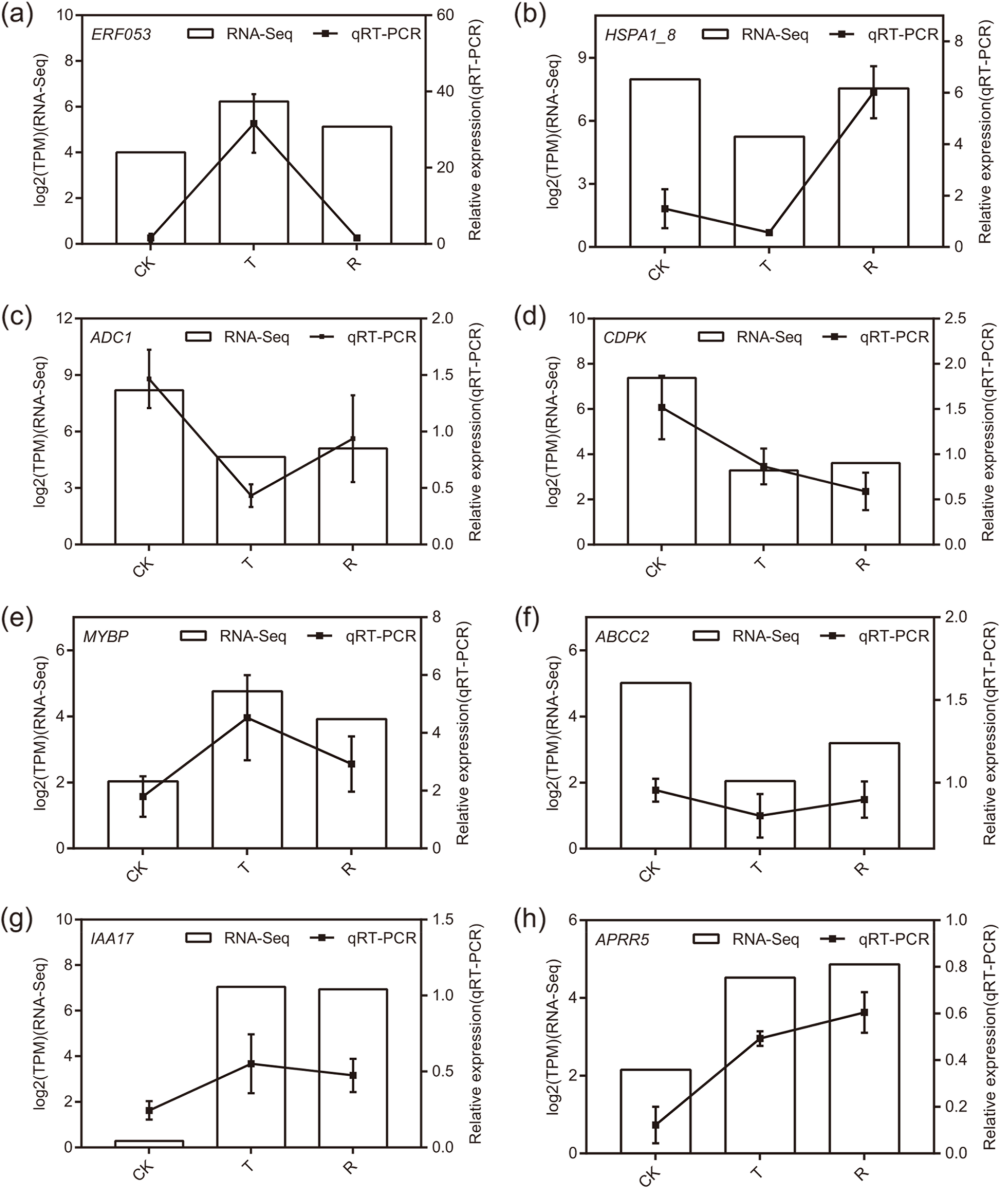
qRTPCR confirmation of the relative expression levels of transcripts in *Iris lactea* var. *chinensis* under normal watering (CK), water-stressed (T), and rehydration -treated (R) condition. Relative expression levels in transcript abundance obtained by both qRT- PCR and RNA-Seq are presented for eight different genes (**a**) *ERF053*, (**b**) *HSPA1_8*, (**c**) *ADC1*, (**d**) *CDPK*, (**e**) *MYBP*, (**f**) *ABCC2*, (**g**) *IAA17*, and (**h**) *APRR5*. The signal intensity of each transcript was normalized using *Actin11*. The y-axis shows the normalized expression level of the transcript

To validate the reliability of the transcriptomic data, eight candidate genes, namely, *ERF053*, *HSPA1_8*, *ADC1*, *CDPK*, *MYBP*, *ABCC2*, *IAA17* and *APRR5*, were randomly selected for qRT-PCR analysis. The results showed that the expression levels of eight candidate genes in the three samples of plants under water-stressed, plants that were rehydrated or plants that were well watered were similar to the RNA-seq data (Fig. 3). Together, these data showed that the RNA-seq analysis was reliable.

### Detection of differentially expressed genes (DEGs)

**Fig. 4:**
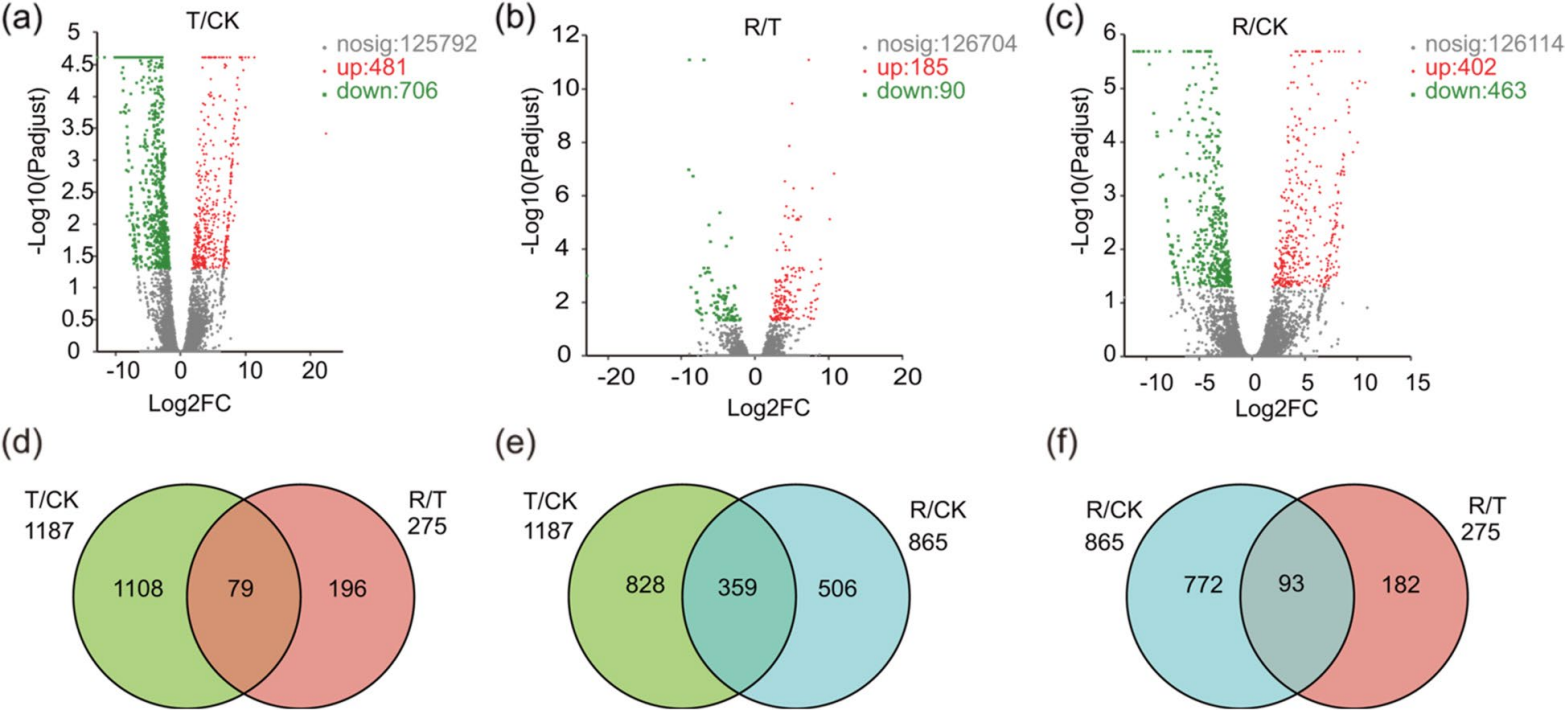
Expression profiling of differentially expressed genes (DEGs) in *Iris lactea* var. *chinensis* under normal watering (CK), water-stressed (T), and rehydration -treated (R) condition. Scatter plot showing DEGs in the (**a**) T/CK, (**b**) R/T, and (**c**) R/CK comparison groups. Venn diagrams showing the overlap between the DEGs identified of (**d**) T/CK and R/T, (**e**) T/CK and R/CK, (**f**) R/CK and R/T comparison groups

To evaluate drought tolerance of *I. lactea* var. *chinensis*, the DEGs in the three samples were visualized as scatterplots. In total, 1187 (Fig. 4a, Additional file 2: Table S2), 275 (Fig. 4b, Additional file 3: Table S3), and 865 (Fig. 4c, Additional file 4: Table S4) DEGs according to |log_2_(fold change (FC))| > = 1 and *P*-adjust < 0.05 were detected in the T/CK, R*/*T, and R*/*CK comparison groups, respectively. There were 481, 185, and 402 upregulated DEG numbers in the three comparison groups (T/CK, R*/*T, and R*/*CK), respectively, and the corresponding numbers of downregulated DEGs numbers were 706, 90, and 463, respectively. In the T*/*CK comparison group, there were more downregulated DEGs than upregulated DEGs, indicating that water-stressed inhibited global gene expression (Fig. 4a, Additional file 2: Table S2).

Venn diagrams showing the number of upregulated DEGs and downregulated DEGs in each comparison (three ways) are shown in Additional file 5: Fig. S1. Interestingly, 73 genes were found to be common between the T/CK and R/T groups, but not the R/CK group, indicating that these genes expressed in the rehydrated plants were virtually the same as those in the CK plants (*P*-adjust > 0.05). Among the 73 DEGs, 29 DEGs were upregulated in T/CK and downregulated in R/T (Additional file 5: Fig. S1c, Additional file 6: Table S5), while 44 DEGs were downregulated in T/CK and upregulated in R/T (Additional file 5: Fig. S1g, Additional file 7: Table S6). These data showed that these 73 DEGs may exhibit high plasticity under plant water-stressed and rehydration. For example, the gene related to primary-amine oxidase (*PAO*; TRINITY_DN60247_c0_g2) expression was upregulated 32.7-fold in response to water-stressed and downregulated to the CK level after rehydration. Similarly, the expression of the gene related to germacrene D synthase (*GDS*; TRINITY_DN45420_c1_g3) increased 655.6-fold after water-stressed treatment and then decreased after rewatering. In contrast, the expression of two *UGT85A* genes (TRINITY_DN44873_c0_g4 and TRINITY_DN53280_c0_g1) were downregulated under water-stressed but upregulated during the water recovery period. Additionally, two-way Venn diagrams were constructed that illustrate the overlap between the DEGs identified in the CK, drought, water recovery treatments, as shown in Fig. 4d, e and f, the results of which are similar to those of the report of Zhang et al. [30].

### GO functional and pathway enrichment analysis of DEGs

**Fig. 5:**
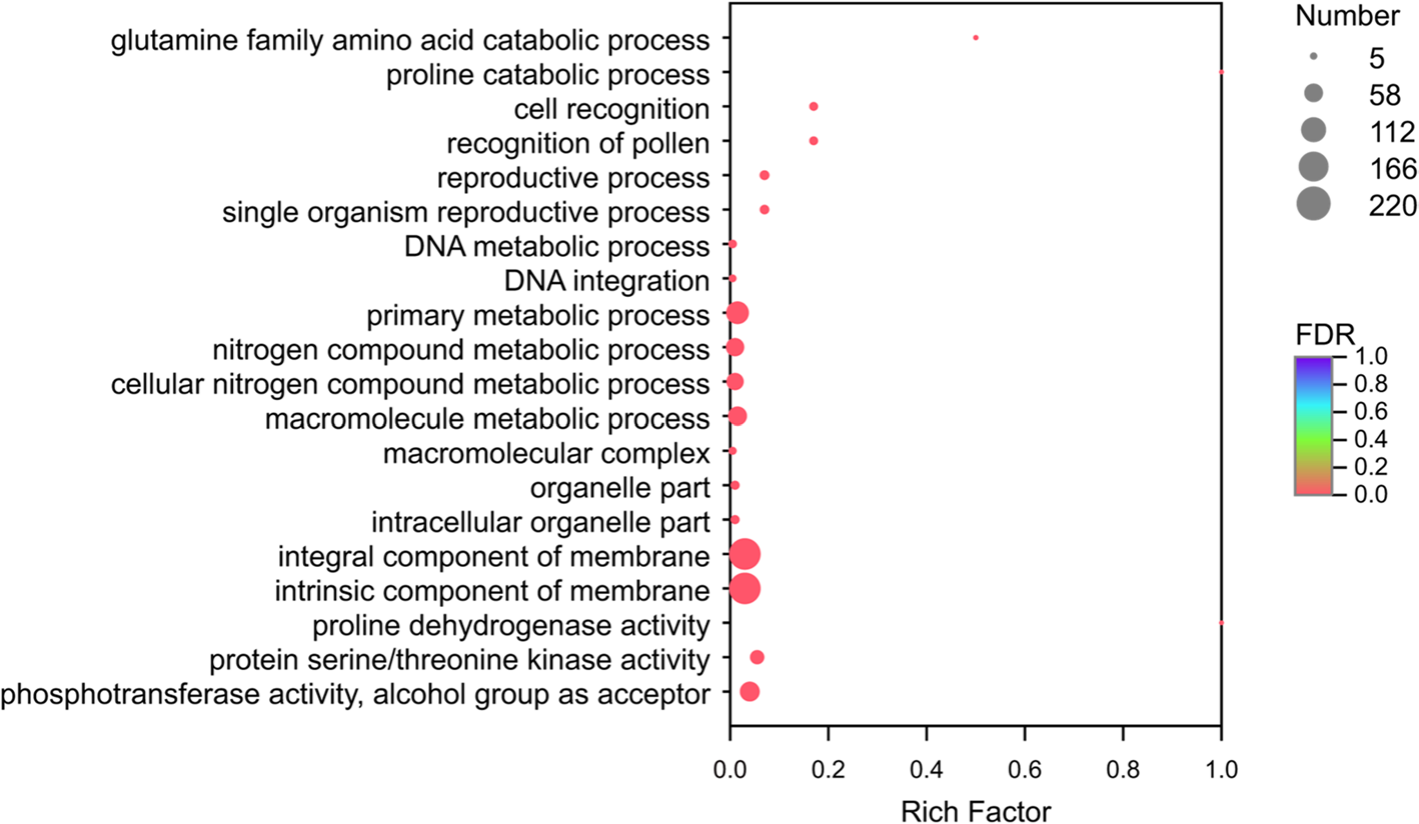
Gene ontology (GO) of DEGs in *Iris lactea* var. *chinensis* under drought stress

To elucidate the potential mechanism of the drought tolerance of *I. lactea* var. *chinensis*, the DEGs were mapped against the information within the GO database and subjected to enrichment analysis, the results of which were classified into three major functional categories based on the criteria of *P*-adjust< 0.05. In the comparison of the water-stressed treatment and the CK, 42 GO terms were significantly enriched in the biological process (BP) category. For example, the DEGs involved in the amino acid biosynthetic and metabolic processes were associated with the ‘glutamine family amino acid catabolic process’, ‘proline catabolic process’, ‘amide biosynthetic process’, and ‘cellular amide metabolic process’; those associated with recognition included ‘cell recognition’ and the ‘recognition of pollen’; those associated with the organic substance metabolic process included the ‘nitrogen compound metabolic process’, ‘cellular aromatic compound metabolic process’, and ‘heterocyclic metabolic process’; and the DEGs related to the biosynthetic and metabolic process of nucleic acids and proteins were associated with the ‘DNA metabolic process’, ‘RNA biosynthetic process’, ‘peptide biosynthetic process’, and ‘cellular protein metabolic process’. Additionally, the identified expected DEGs related to the response to various other types of abiotic stresses were associated with the ‘cellular response to osmotic stress’, ‘cellular response to salt stress’, and ‘cellular response to blue light’ (Fig. 5, Additional file 8: Table S7, Additional file 9: Tables S8, and Additional file 10: Table S9). For the cellular component (CC) category, only 15 GO terms were significantly enriched in the comparison of the drought-stressed and CK plants; these DEGs were mainly related to the components of membrane cytoplasmic part and organelle part (Fig. 5). For the molecular function (MF) category, genes were primarily enriched in various types of proteinase activity, including proline dehydrogenase activity, protein kinase activity and transferase activity (Fig. 5). Nonetheless, we found that only four and eight GO terms were significantly enriched in the R/T (Additional file 9: Table S8) and R/CK groups (Additional file 10: Table S9), respectively.

To reveal the underlying mechanism of *I. lactea* var. *chinensis* tolerance to drought stress, pathway enrichment analyses of the DEGs based on the KEGG database were performed. In the T/CK group, 64 DEGs were significantly enriched in six pathways (*P*-adjust < 0.05), including ‘plant-pathogen interaction’, ‘alpha-linolenic acid metabolism’, ‘circadian rhythm-plant’, ‘ABC transporters’, ‘arginine and proline metabolism’, etc. (Table 2). Some of the 64 DEGs play an important role in plant drought tolerance [31–33]. For example, the receptor-like kinases-encoding gene *BAK1/SERK1* (TRINITY_DN60727_c4_g2), hydroperoxide dehydratase-encoding gene *AOS* (TRINITY_DN51813_c4_g1), and pseudoresponse regulator-encoding gene *PRR5* (TRINITY_DN56996_c1_g3, TRINITY_DN62580_c5_g2, TRINITY_DN51806_c4_g2, and TRINITY_DN56996_c1_g1) were upregulated in the T/CK comparison group. The whole report for the DEGs in each comparison group is shown across Additional file 11: Table S10, Additional file 12: Table S11 and Additional file 13: Table S12.

**Table 2:** Expression profiling of DEGs in *Iris lactea* var*. chinensis* significantly enriched pathways under drought stress (*P*-adjust< 0.05)

Pathway ID	Description	*P* value	*P*-adjust
ko04626	Plant-pathogen interaction	1.19805E-06	8.39E-05
ko00592	alpha-Linolenic acid metabolism	1.13685E-05	0.00039
ko04712	Circadian rhythm-plant	0.00053171	0.01240
ko02010	ABC transporters	0.00122627	0.02146
ko00330	Arginine and proline metabolism	0.00382203	0.04459
ko00480	Glutathione metabolism	0.00356642	0.04993
ko04075	Plant hormone signal transduction	0.0079252	0.07925
ko04146	Peroxisome	0.01387324	0.12139
ko04016	MAPK signaling pathway-plant	0.03042858	0.23666
ko04141	Protein processing in endoplasmic reticulum	0.03785114	0.26495

### TFs, transporter proteins (TPs), ROS-scavenging systems, and proline metabolism affect the plant response to drought stress

**Fig. 6:**
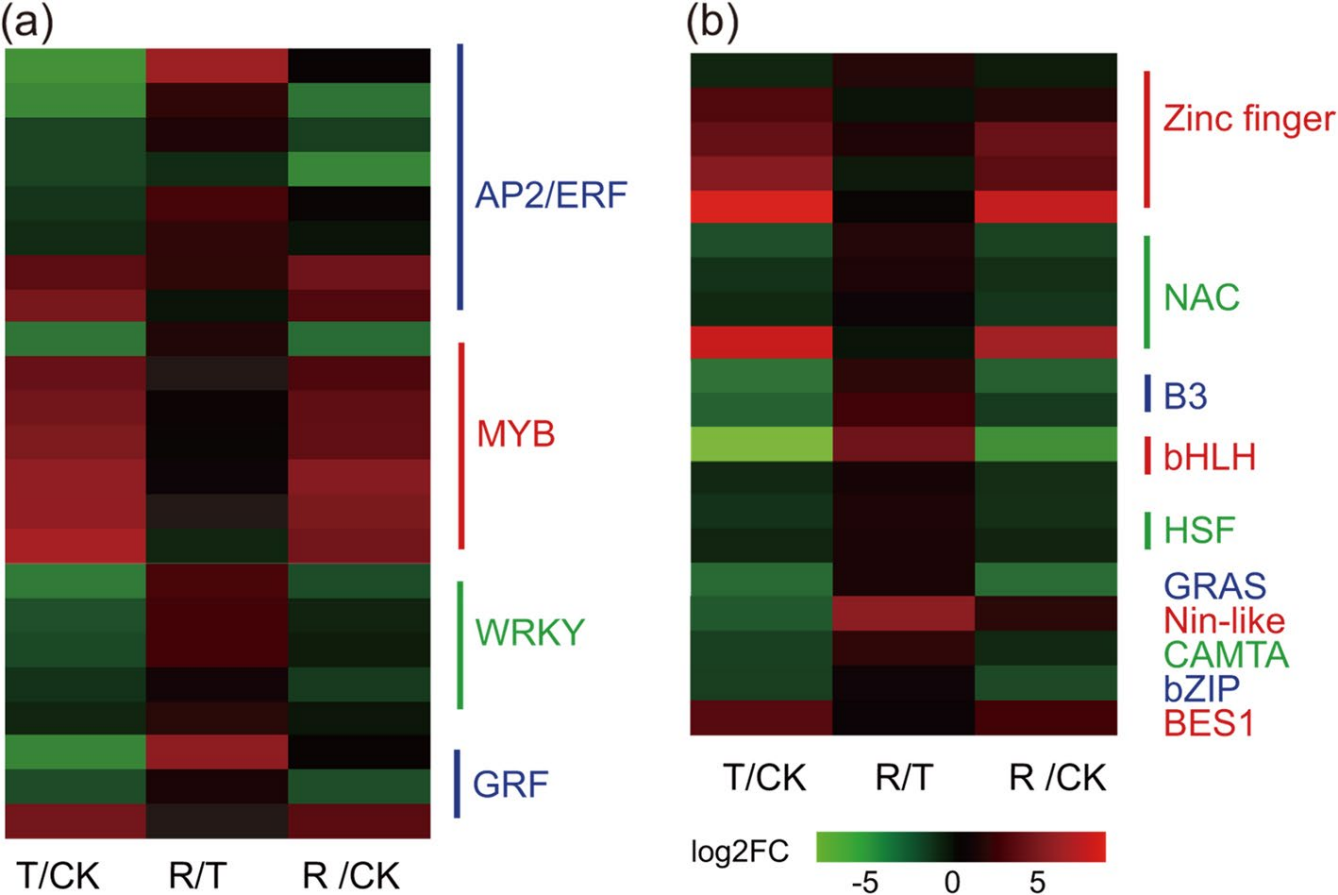
Expression patterns of drought-regulated differentially expressed genes (DEGs) of transcription factors (TF) genes between different treatment: normal watering (CK), water-stressed (T), and rehydration -treated (R)

**TFs.** From our database searches, at least 43 DEGs encoding TFs were found (Additional file 14: Table S13), which were visualized as the heatmap (Fig. 6). The TFs were divided into 14 subfamilies, which included APETALA2/ethylene-responsive (AP2/ERF), MYB, WRKY, zinc finger, NAC, and growth-regulating factors (GRF) TFs, etc. As shown in Fig. 6, most DEGs encoding TFs exhibited a downregulated expression in plants under water-stressed compared to the CK plants, which is consistent with the aforementioned DEGs (Figs. 4 and 6). However, some DEGs encoding TFs exhibited upregulated expression in the water-stressed treatment; e.g., two related genes encoding AP2/ERF TFs (TRINITY_DN59365_c4_g7, TRINITY_DN48541_c0_g3), six related genes encoding MYB TFs (TRINITY_DN58225_c1_g1, TRINITY_DN63921_c6_g1, TRINITY_DN56464_c2_g2, TRINITY_DN57679_c2_g2, TRINITY_DN43447_c3_g3 and TRINITY_DN44333_c4_g3) and four related genes encoding zinc finger TFs (TRINITY_DN59156_c3_g4, TRINITY_DN64408_c4_g1, TRINITY_DN44819_c3_g3 and TRINITY_DN57150_c6_g1) were significantly upregulated in the water-stressed treatment. In the R/CK group, most gene expression levels were similar to those in the T/CK group, suggesting that rehydration might affect the expression of a small amount genes, which was validated by the gene expression level in the R/T comparsion group (Fig. 6).

**Fig. 7:**
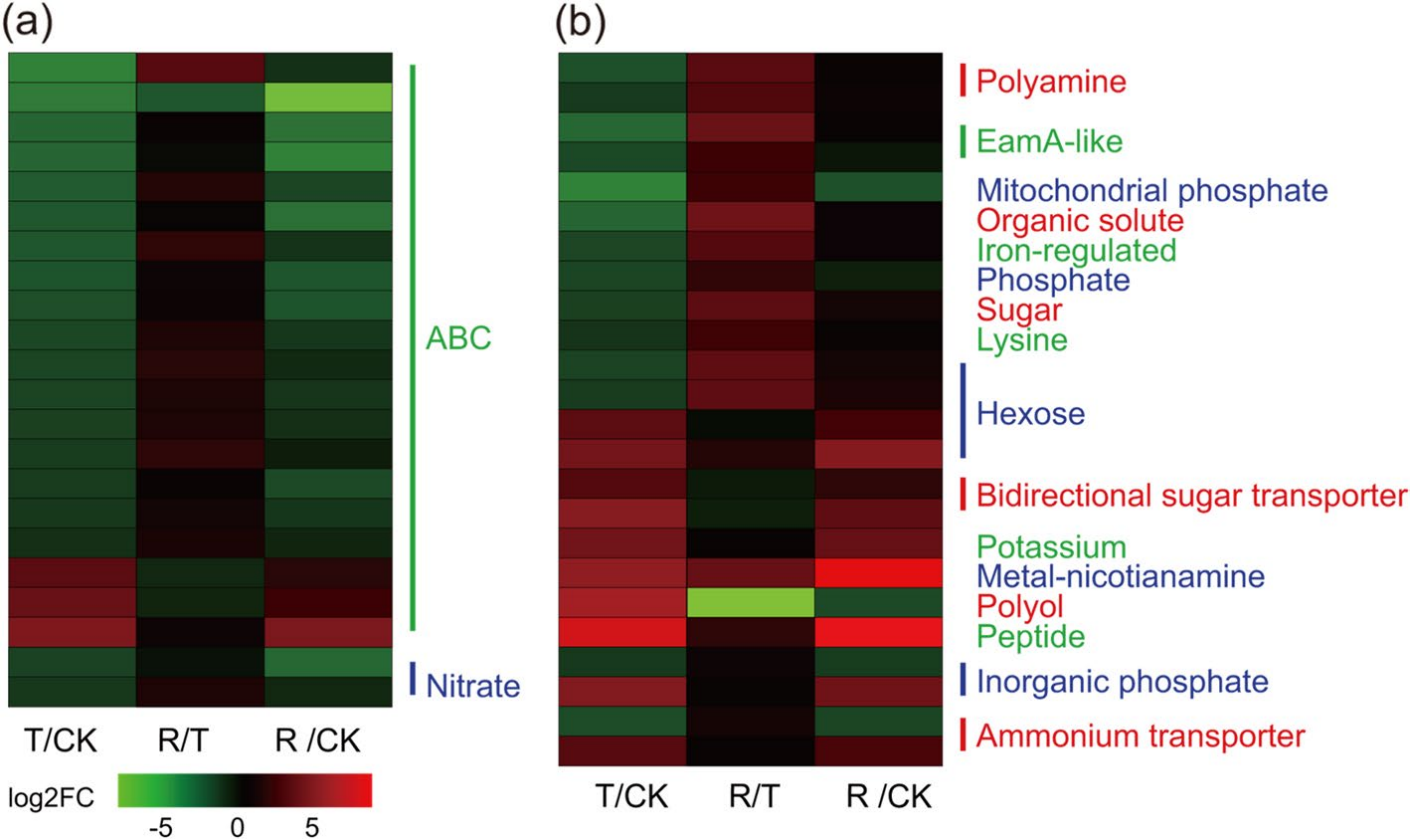
Expression patterns of drought-regulated differentially expressed genes of transporter genes between different treatment: normal watering (CK), water-stressed (T), and rehydration -treated (R)

**TPs.** Forty-six DEGs encoding transporters were detected in the RNA-seq data, including those encoding ABC, nitrate, hexose, polyamine transporters (Fig. 7, Additional file 15: Table S14). In the T/CK and R/CK groups, most DEGs were downregulated, similar to the DEGs related to TFs. There were some upregulated under water-stressed, e.g., two genes related to bidirectional sugar transporters (TRINITY_DN50598_c1_g2, TRINITY_DN44794_c7_g1), one gene related to potassium transporter (TRINITY_DN45507_c4_g1), one gene related to metal-nicotianamine transporters (TRINITY_DN46504_c5_g1), one gene related to polyol transporters (TRINITY_DN52400_c0_g1). However, rehydration can affect the marked changes in expression of these DEGs, as shown in the R/T comparsion group (Fig. 7).

**Fig. 8:**
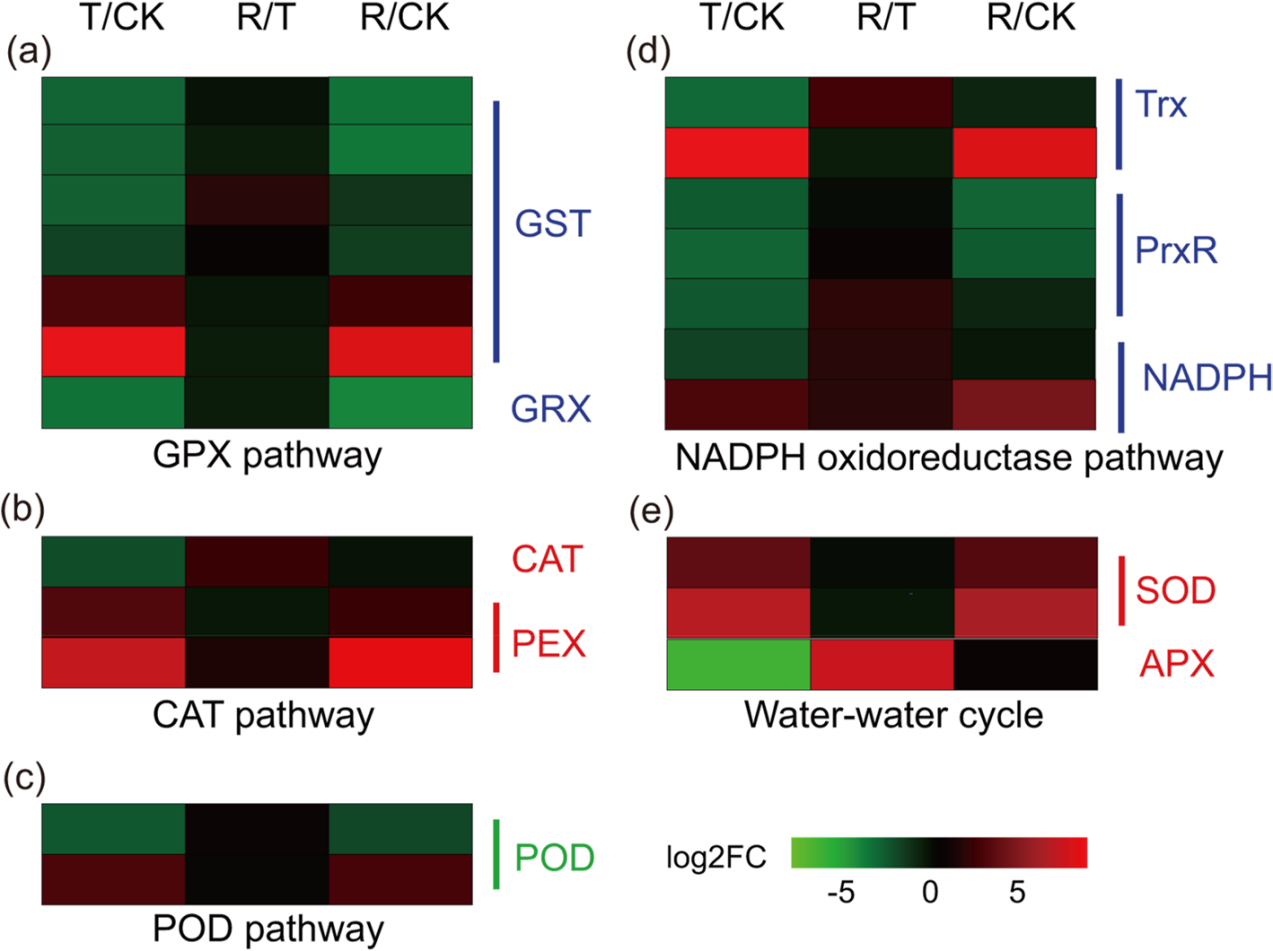
Expression patterns of drought-regulated differentially expressed genes (DEGs) encoding enzymes related to the reactive oxygen species (ROS) scavenging system categorized as being involved in the (**a**) glutathione peroxidase (GPX) pathway, (**b**) catalase (CAT) pathway, (**c**) peroxidase (POD) pathway, (**d**) NADPH oxidoreductase pathway and (**e**) water-water cycle between different treatment: normal watering (CK), water-stressed (T), and rehydration -treated (R)

**Fig. 9:**
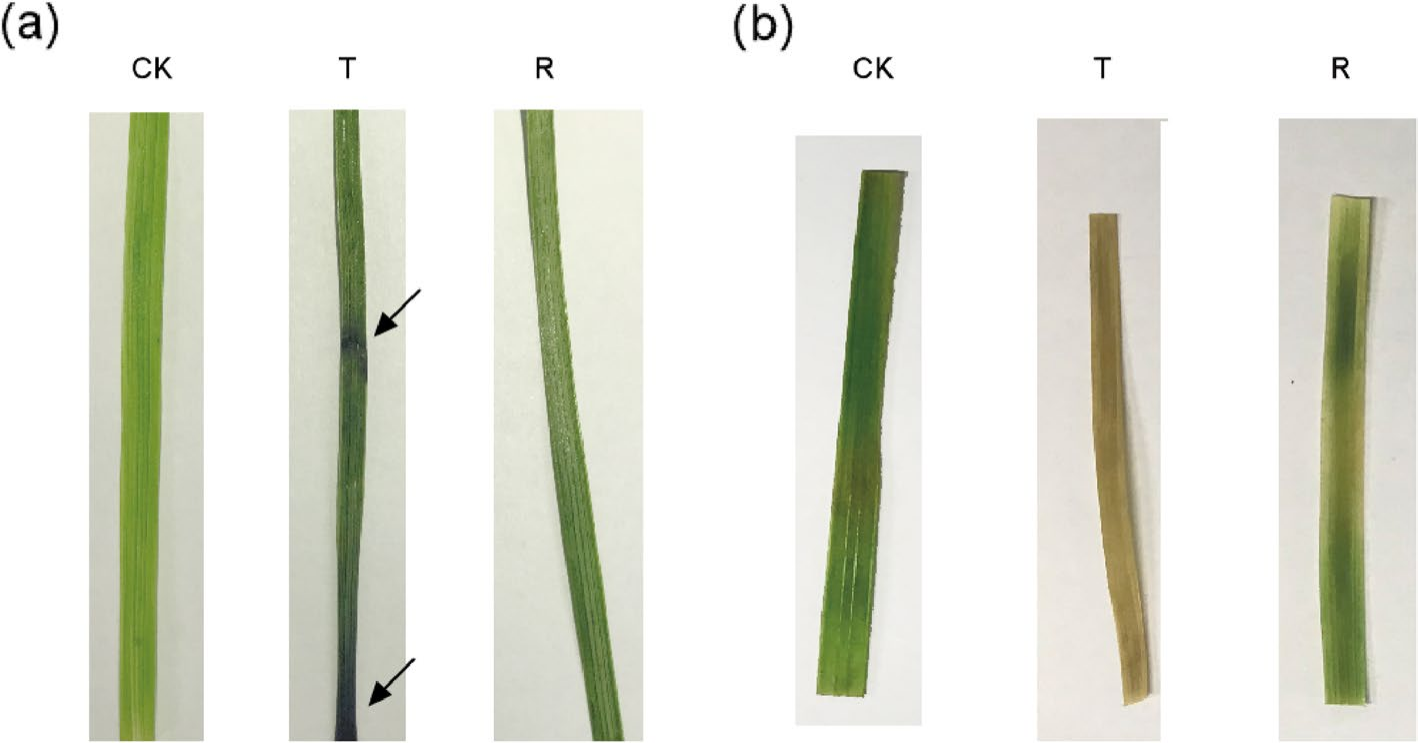
Comparison of O_2_^·-^ and H_2_O_2_ accumulation in *Iris lactea* var. *chinensis* leaves under normal watering (CK), water-stressed (T), and rehydration -treated (R) condition. **a** NBT staining, **b** DAB staining. Arrow indicate the location of O_2_^·-^ and H_2_O_2_ accumulation

**ROS-scavenging system.** In only the T/CK comparison group, there were 22 DEGs encoding ROS-scavenging enzymes, which were categorized as being involved in the glutathione peroxidase (GPX) pathway, catalase (CAT) pathway, peroxidase (POD) pathway, NADPH oxidoreductase pathway and water-water cycle (Fig. 8, Additional file 16: Table S15). Among them, the GPX pathway contained the most DEGs involved in the ROS-scavenging system, including glutathione S-transferases (*GST*s) and glutaredoxin (*GRX*). Under water-stressed, one *GRX* (TRINITY_DN47416_c1_g1) and four *GST*s (TRINITY_DN53475_c0_g3, TRINITY_DN52312_c2_g1, TRINITY_DN62418_c3_g3, and TRINITY_DN53475_c0_g2) had a significantly downregulated expression and only two *GST*s (TRINITY_DN67517_c0_g1 and TRINITY_DN47288_c0_g1) were significantly upregulated. In the NADPH oxidoreductase pathway, most DEGs that encoded three key enzymes, viz., thioredoxin (*Trx*), NAD(P)H-quinine oxidoreductase, and peroxiredoxin (*PrxR*), were downregulated (Fig. 8). Additionally, two *SOD*s (TRINITY_DN58115_c0_g1, TRINITY_DN57370_c0_g2) were significantly upregulated expression under water-stressed. In addition, these DEGs were associated with two *POD*s, two *PEX*s, one *CAT*, and one *APX*, which possibly are involved in the plant response to drought stress (Fig. 8). After rehydration, the expression level of 22 DEGs recovered to that of the CK. To verify the in situ the accumulation of H_2_O_2_ and O_2_^·-^ under water-stressed treatment, histochemical assays with diaminobenzidine (DAB) and nitroblue tetrazolium (NBT) were used to quantify ROS activity. After the water-stressed treatment, dark blue spots (stained with DAB; Fig. 9a) and brown spots (stained with NBT; Fig. 9b) were deposited in the *I. lactea* var. *chinensis* leaves. After rehydration treatment, the deposited blue spots (stained with DAB; Fig. 9a) and brown spots (stained with NBT; Fig. 9b) turned to lighter in color. The histochemical experiment further demonstrated that ROS accumulation was involved in the drought stress of *I. lactea* var. *chinensis*.

**Fig. 10:**
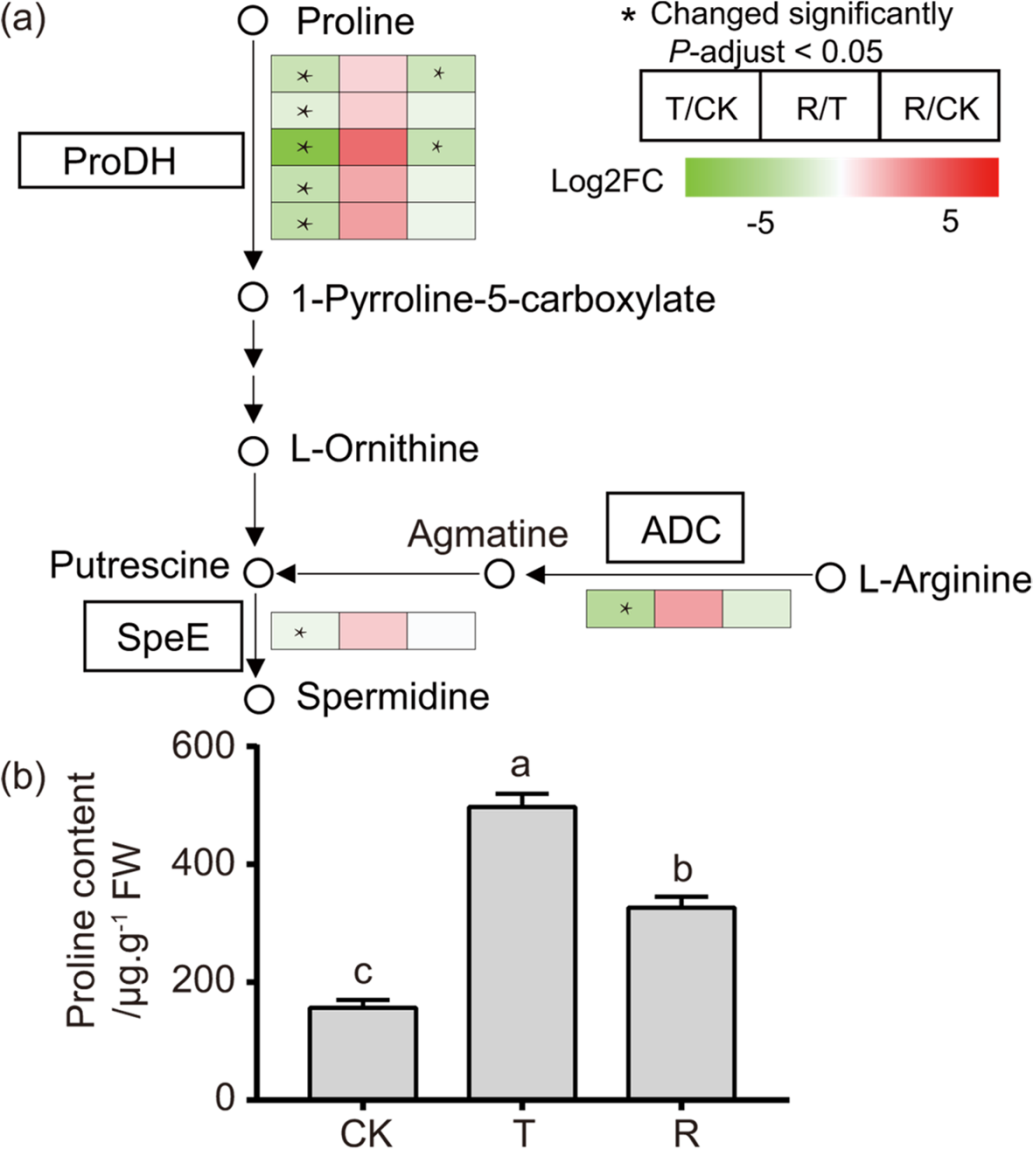
**a** Gene expression of the proline and glutathione metabolism pathway and the response in *Iris lactea* var. *chinensis* under the treatment of normal watering (CK), water-stressed (T), and rehydration -treated (R). Metabolites detected shown in bold, solid lines represent one-step reactions, and dashed lines represent multi-step reactions. The abbreviations in boxes indicate enzymes catalyzing the reactions. The expression patterns of unigenes encoding these enzymes under drought stress and rehydration compared to the controlsare shown in the color boxes. The small boxes in the left, middle and right sides of each colored box indicate altered levels between T/CK, R/T and R/CK, respectively. Significantly altered expression (*P*-adjust < 0.05) is indicated by an asterisk in the boxes. Abbreviations for enzymes: ProDH: proline dehydrogenase; speE: spermidine synthase; ADC: Arginine decarboxylase. **b** Comparison of proline concentration in *Iris lactea* var. *chinensis* under normal watering (CK), water-stressed (T), and rehydration -treated (R) condition

**Proline metabolism** Proline dehydrogenase (ProDH), a mitochondrial enzyme, is a key enzyme in the first step of the proline metabolic process (Fig. 10a). Under water-stressed, *ProDH* expression in *I. lactea* var. *chinensis* plants was downregulated. When the plants were rehydrated, the *ProDH* expression level increased compared to that in the plants under the water-stressed treatment (Fig. 10a, Additional file 17: Table S16). The genes located downstream of the proline catabolism process, including *ADC* and *SpeE*, were downregulated under drought stress (Fig. 10a, Additional file 17: Table S16). The free proline content was consistent with the results of the transcriptome analysis. After water-stressed, the *I. lactea* var. *chinensis* plants’ free proline content sharply increased to 497.3 μg/g FW (*p* < 0.05), which was 3.17-fold to the level of CK plants (Fig. 10b). And then after rehydration treatment, free proline content decreased to 326.9 μg/g FW, which was significantly decreased by 34.27% compared to that under water-stressed treatment (p < 0.05) (Fig. 10b). These data suggest that proline can accumulate in *I. lactea* var. *chinensis* plants under drought stress, consistent with previous reports of water-stressed plants.

## Discussion

Drought has spread to many regions because of ongoing global climate change and plants have evolved many elaborate mechanisms to adapt to drought stress. *I. lactea* var. *chinensis* is an ecologically important herbaceous species, and exhibits strong phenotypic plasticity, enabling these plants to grow in many types of habitats, including deserts. Previous studies on *I. lactea* var. *chinensis* have mainly focused on its phytoremediation capability, salt tolerance, heavy metal ion tolerance and PEG-mediated drought tolerance traits [26, 30, 31, 34]. Nonetheless, the underlying mechanism of *I. lactea* var. *chinensis* tolerance to direct water-stressed (severe dehydration or withholding of water), needs to be explored. In the present study, RNA-seq technology was used for transcriptome profiling of *I. lactea* var. *chinensis* under direct water-stressed to characterize the molecular mechanism of plant drought tolerance.

As major regulator of target gene expression, TFs play important roles in the plant response to abiotic stresses by binding to cis-regulatory elements in the promoter region of different stress-related genes [35], mediating both ABA-dependent and ABA-independent signal transduction pathways [6]. In the ABA-dependent pathway, we detected that 6/7 MYBs and 1/4 NACs were upregulated (Fig. 6, Additional file 14: Table S13), which regulate stress-responsive genes that are involved in the protection of cell integrity and plant development under stress. With 100–200 family members commonly found in plant species [36], the MYB family plays crucial roles in regulating plant growth and development, metabolite synthesis, phytohormones, and signal transduction, especially in response to abiotic stress [37]. In addition, MYB TFs which specifically expressed in guard cells, can affect the stomatal movement when suffering various stress responses in many model plant species [38]. The *AtMYB41* gene in *Arabidopsis* has been proven to be involved in cell expansion and cuticle deposition under drought stress [39]. *AtMYB60*, an R2R3-MYB gene of *Arabidopsis* that is specifically expressed in guard cells to reduce stomatal opening during drought [40]. In ABA-independent pathways, we detected 2/8 AP2/ERFs that regulate the expression of different drought-inducible genes (Fig. 6, Additional file 14: Table S13). The AP2/ERF family is a large group of plant-specific TFs involved in plant abiotic stress responses and various biological and developmental processes [41]. Overexpression of ERF-related genes in *Zea mays* [42], *Glycine max* [43], and *Gossypium hirsutum* [44] has been shown to enhance the ability of plants to tolerate drought. In addition, studies have shown that ERF can act as an on-off switch maintaining ROS detoxification of H_2_O_2_ homeostasis in response to different stresses [45]. ERF3 can bind to the cis-element as-1 in response to oxidative stress, and regulate the expression of SOD to reduce the level of ROS in plants, improving the ability to withstand drought stress [46].

It has been reported that metal-nicotianamine transporters probably acts as transporters of iron- and metal-nicotianamine chelates [47]. Increased transport of polyols in both the phloem and the xylem occurs frequently as a result of drought stress [48]. In this study, several genes encoding bidirectional sugar transporters, metal-nicotianamine transporters, and polyol transporters were upregulated after drought (Fig. 7, Additional file 15: Table S14), which indicated that *I. lactea* var. *chinensis* drought tolerance is related to sugar, potassium transport, and iron transport. Therefore, these results showed that drought tolerance of *I. lactea* var. *chinensis* possibly depends on transporter-encoding gene expression changes.

In general, abiotic stresses cause ROS to accumulate, leading to the inhibition of plant growth [49]. Therefore, it is important to scavenge ROS to protect cells from oxidative damage through antioxidant and antioxidant enzymes. In the present study, six *GST*s were associated with *I. lactea* var. *chinensis* drought tolerance (Figs. 8 and 10, Additional file 16: Table S15). Interestingly, a recent report showed that *GST*s likely participated in *I. lactea* var. *chinensis* salt tolerance [25]. These results suggest that the *GST*s found to be involved in drought and salt tolerance by regulating their ROS-scavenging level. Additionally, two *SOD*s were upregulated in response to drought stress (Additional file 16: Table S15); the enzymes catalyze the disproportionation of O_2_ˉ into H_2_O_2_ and O_2_, after which the H_2_O_2_ is converted back into H_2_O (catalyzed by APX) in the water-water cycle [50], which was also found in several plant species, such as *Ipomoea batatas* [51], *Oryza sativa* [52], and *Lens culinaris* [53]*.*

Proline, an amino acid, plays an important role as osmolyte for osmotic adjustment and as a reservoir of carbon and nitrogen and has been shown to protect plants against radical-induced damage [6, 54, 55]. Proline accumulation is a common physiological response to drought stresses in many plant species [56–58]. In this study, there were several GO terms and KEGG pathways involved in proline metabolism in the comparison of plants under drought stress and the CK (Fig. 10, Additional file 17: Table S16). In plants, the precursor substance glutamate is synthesized to proline by two enzymes, pyrroline-5-carboxylate synthase (P5CS) and pyrroline-5-carboxylate reductase (P5CR) [59]. By inducing the expression of *P5CS* to increase proline concentration, Su and Wu [60] reported that *O. sativa* can tolerate increased salt and water-stressed. We also observed an increased expression of *P5CS* under drought stress to promote proline accumulation. On the other hand, the proline degradation is a reversible reaction which catalyzed by *ProDH* and P5C dehydrogenase (*P5CDH*) [61]. The *I. lactea* var. *chinensis ProDH* expression was significantly downregulated under drought stress, indicating that the catabolism of proline was inhibited so that it could accumulate (Fig. 10). In line with these results, *ProDH* expression in *Arabidopsis* was shown to be downregulated in response to dehydration and upregulated after rehydration [62]. Therefore, the change in proline metabolism could be beneficial for proline accumulation for drought tolerance of *I. lactea* var. *chinensis* through two main biochemical pathways: the first pathway involves promoting the synthesis of proline through increased expression of *P5CS*, and the second pathway involves inhibiting the degradation of proline through decreased expression of *ProDH*.

Plants often suffer multiple abiotic stresses simultaneously, and overlapping signals and pathways may contribute to plant stress resistance [63]. Combining the previously investigated mechanisms of *I. lactea* var. *chinensis* underlying abiotic stress resistance to toxic heavy metal [24], salt [25, 26], and PEG-mediated drought [26], TFs and the ROS detoxification system were found to mitigate the effects of all the above stresses. However, they may be associated with unique signal transduction and response mechanisms against different types of abiotic stress. For example, transporter activity, transmembrane transporter activity and the membrane were coregulated under both NaCl and PEG stresses associated with different regulatory strategies, suggesting that the same genes play different roles under different stresses. These genes might be the potentially useful genes in understanding the mechanism of *I. lactea* var. *chinensis* for multiple abiotic stresses resistance.

*I. lactea* var. *chinensis* can survive in arid and semiarid areas where there is always intermittent drought and/or rewetting events [64]. Previous studies have shown that recovery is as important as the stress treatment is for plant drought adaptation, as efficient recovery affects further plant growth and development [65]. Rehydration could lead to a compensatory growth effect, including an increase in the plant height [66], root length, leaf area, number of leaves [67], and rate of dry matter accumulation [68]. However, rare research involving omics methods has focused on rehydration mechanisms, which are thus far largely uncharacterized. Talame et al. [69] compared the expression changes in leaves of barley plants during drought-stress and rehydration treatments, and found a very low overlap of differentially regulated transcripts. The rehydration-inducible genes identified may be involved with cytochrome P450, which is involved in xenobiotic detoxification, ABA degradation [70], and the recovery process from dehydration-induced damage (protein degradation, ROS-scavenging enzymes, and regulatory proteins), suggesting that regulation during the recovery from drought may involve regulation at both the transcriptional and the posttranscriptional levels. Ford et al. [71] tested the responses of drought-tolerant wheat cultivars during rewatering after water-stressed treatment. The abundance of the most drought-tolerant cultivar’s glycolysis enzymes increased sharply, indicating the need for energy during the recovery phase. In the present study, 73 DEGs were found to be common between T/CK and R/T groups but not R/CK group (Additional file 5: Fig. S1, Additional file 2: Table S2, Additional file 3: Table S3 and Additional file 4: Table S4). Their expression exhibited a reversible trend during water-stressed and the rehydration period. However, the functions of these genes are unknown, these findings of which are similar to those of Talame et al. [69]. Moreover, several genes could be the focus of future research. For example, a primary-amine oxidase (*PAO*)-related gene was more highly expressed in the both drought-stressed plants and rehydrated plants than in the CK plants. It has been reported that the *MmPAO2* gene is highly responsive to drought stress [72]. Moreover, as components of plant secondary metabolic pathways, sesquiterpenoids are involved in many biological properties [73], and have been shown to participate in the response to drought stress in *Salvia dolomitica* [74]. The gene encoding germacrene D synthase (GDS), a main enzyme in the biosynthesis of sesquiterpenoids, was upregulated in *I. lactea* var. *chinensis* in response to drought stress and downregulated in the rehydration treatment (Additional file 6: Table S5). In plants, UDP-glycosyltransferases (UGTs) glycosylate various phytohormones and metabolites in response to abiotic stress. For example, the expression levels of *UGT85A1* and *UGT85A2* were shown to be downregulated under drought stress in an *Arabidopsis* mutant [75]. In line with these results, our RNA-seq data revealed that two genes related to *UGT85A* expression were downregulated during drought stress and upregulated during the water recovery period (Additional file 3: Table S3).

## Materials and methods

### Plant materials, growth conditions and water-stressed treatments

Seeds of *I. lactea* var. *chinensis* were collected from the Ordos in Inner Mongolia, China, in the early autumn. Seeds were sown in a seedling tray. After 30 days, seedlings that were about 10 cm tall were transferred to plastic pots (10 cm diameter and, 10 cm height) with commercial peat substrate. *I. lactea* var. *chinensis* plants were grown in a greenhouse at the Chinese Academy of Agricultural Sciences with a natural photoperiod, a daily temperature between 32 °C (daytime) and 25 °C (night) and a relative humidity of approximately 60%. Plants were well irrigated from days 0 to 30 prior to the start water-stressed treatments (Fig. 1).

The seedlings were divided into three groups to be used as the control (CK, normal watering), water-stressed samples (T) and rehydration-treated samples (R). The water-stressed samples were naturally air-dried from days 31 to 37 after transplanting them. At this time, about 75% of the leaves showed wilting. For the rehydration -treated samples (R), the water supply was restored from days 38 to 40 (3 days treatment) after they had been water stressed (Fig. 1). On the day 41, the whole plant were collected from CK, T and R groups at the same time to measure the phenotypic characterization, free proline concentration, ROS accumulation and RNA extraction, respectively.

### Phenotypic characterization and the relative water content

The plant height, root length, and root/shoot ratio were measured respectively. The relative water content (RWC) of aerial parts of *I. lactea* var. *chinensis* was measured using the method of Parida et al. [76]. The dry weight resulting from each treatment was obtained by drying at 80 °C for 8 h. RWC was calculated using the following formula: RWC (%) = (FW-DW)/FW × 100 (FW: fresh weight and, DW: dry weight).

#### Determination of proline concentration and ROS accumulation

Three treatment’s fresh leaves proline concentration and ROS accumulation were quantization respectively. Proline concentration was determined with acid ninhydrin reagent followed by Bates et al. [77] methods and formula: μmoles proline/g of fresh weight material = [(μg proline/ml × ml toluene) / 115.5 μg/μmole]/[(g sample)/5]. The accumulation of H_2_O_2_ and O_2_^·-^ production was quantization the ROS activity. H_2_O_2_ and O_2_^·-^ was histochemical detection with diaminobenzidine (DAB) and nitroblue tetrazolium (NBT), respectively followed by Romero-Puertas et al. [78].

### RNA isolation, cDNA library construction and sequencing

For each treatment, 30 samples were randomly divided into 3 groups as 3 biological replicates. For each biological replicate (10 individuals), total RNA was extracted from all the overall plant using the TRIzol reagent (Invitrogen, Carlsbad, CA, USA) according to the manufacturer’s protocol. After total RNA extraction, reverse transcription reactions were performed with the High Capacity cDNA Reverse Transcription Kit (Applied Biosystems, Foster City, CA, USA) with following DNase I treatment. The poly (A) mRNA was isolated using magnetic beads with Oligo (dT). Then fragmented into short fragments of 200 ~ 700 bp after mixing with the fragmentation buffer. Then cDNA was synthesized from mRNA fragments and random primers. Short cDNA fragments were purified and dissolved in TE buffer for end repair and single nucleotide A (adenine) addition. After that, the short fragments were ligated with adapters. The suitable fragments were size fractionated for PCR amplification as templates. The sample libraries were used for the quantification and qualification test by Agilent 2100 Bioanaylzer (Agilent Technologies, Palo Alto, CA, USA) and Applied Biosystems StepOnePlus Real-Time PCR System (Applied Biosystems, Foster City, CA, USA). The library products were sequenced using Illumina HiSeq™ 2000 carried out by the Beijing Genomics Institute (BGI), Shenzhen, China.

All raw data were deposited in the National Center for Biotechnology Information (NCBI) and can be accessed in the Short Read Archive (SRA) under the accession number SRP257840.

### De novo assembly and annotation

The raw paired end reads were trimmed and quality controlled by SeqPrep (https://github.com/jstjohn/SeqPrep) and Sickle (https://github.com/najoshi/sickle) with default parameters. Then, clean reads were assembled into contigs using Trinity software [79]. These contigs were subjected to sequence clustering to form longer sequences. Such sequences were defined as unigenes. The Trinity assembly was optimized using TransRate v1.0.3 software of the transcriptome assembly sequence filter [80] and CD-HIT v4.6.8 (http://weizhongli-lab.org/cd-hit/) and the sequence alignment Cluster method were used to remove redundancy and similar sequences, and finally obtain the non-redundant sequence [81]. BUSCO evaluates the assembly integrity of the transcriptome [82]. The sequence assembly quality was evaluated using the number of sequences and bases, GC content, distribution of unigene lengths, average coverage, and N50 statistics [83].

To obtain functional annotation of the assembled unigenes, all of the assembled unigenes were aligned against publicly available databases, including the National Center for Biotechnology Information nonredundant protein (NR, ftp://ftp.ncbi.nlm.nih.gov/blast/db/), the NCBI nucleotide sequence (NT), the Swiss-Prot protein database (SwissProt) [84], Gene Ontology terms (GO) [85], Clusters of Orthologous Groups (COGs) [86], and Kyoto Encyclopedia of Genes and Genomes (KEGG) [87], using the BLASTx algorithm with an E-value threshold of 10^− 5^ [88].

### Analysis of differentially expressed genes (DEGs)

To identify DEGs between two samples, the expression level of each unigene was calculated according to the transcripts per million reads (TPM) method. RSEM [89] was used to quantify gene and isoform abundances. The R statistical package software DESeq2 package [90] was utilized for differential expression analysis. Venn Diagrams were generated using the free online platform of Majorbio Cloud Platform (www.majorbio.com).

Thousands of independent statistical hypothesis tests were separately conducted on DEGs. Then, a *P*-value was obtained, which was corrected using the false discovery rate (FDR) method. Parameters for classifying statistically significant DEGs were as follows: at least a two-fold difference in the transcript abundance (|log_2_FC| > = 1), FC: fold change in expression) and FDR < 0.05(*P*-adjust < 0.05). In addition, functional enrichment analyses, including GO and KEGG database analyses, were performed to identify the GO terms and metabolic pathways in which the DEGs were significantly enriched at a Bonferroni-corrected *P*-value of less than or equal to 0.05 compared with the whole- transcriptome background. GO functional enrichment and KEGG pathway analyses were carried out using Goatools [91] and KOBAS [92].

### qRT-PCR analysis

To validate RNA sequencing’s reliability, 8 DEGs were randomly selected from the above detected DEGs for quantitative analysis of gene expression by RT-PCR. The genes sequences used for primers design were generated from assembled unigenes in the present study. qRT-PCR was performed with ChamQ SYBR Color qPCR Master Mix (2×) (Vazyme Biotech Co., Ltd., Nanjing, China) and BIOER LineGene9600plus Sequence Detector (BIOER, Hangzhou, China), and three replicates were repeated. qRT- PCR was conducted at 95 °C for 5 min, followed by 40 cycles of denaturation at 95 °C for 30 s, annealing at 60 °C for 30 s, and dissociation at 72 °C for 40 s. All primers used for qRT-PCR are listed in Additional file 18: Table S17. The relative expression levels were calculated based on the 2^-ΔΔCT^ method [93]. The *Actin11* was chosen as a reference gene for normalization and primers were generated from *I. lactea* var. *chinensis* partial CDS sequence of *Actin11* (GenBank Accession Number: AB971013).

## Conclusion

In conclusion, we employed the RNA-seq technique to obtain global information on the gene expression of the *I. lactea* var. *chinensis* plants in response to water-stressed and rehydration. Based on the assembled de novo transcriptome, 1187, 275, and 865 DEGs were identified from T/CK, R/T, and R*/*CK comparisons, respectively. GO and KEGG pathway analyses revealed that the DEGs were enriched in several important terms and pathways related to proline metabolism, such as the ‘proline catabolic process’, the ‘proline metabolic process’ and ‘arginine and proline metabolism’. Moreover, many DEGs encoding TFs and transporters, and involved in the ROS-scavenging system were identified. The expression of these 73 DEGs, such as those encoding PAO, GDS, UGT85A, displayed a reversible trend during drought and the rehydration, which may need to be studied in the future. Taken together, our findings showed that drought tolerance of *I. lactea* var. *chinensis* might depend on proline accumulation, the action of TFs and transporters, and a efficient ROS-scavenging system.

## Supplementary Information

**Additional file 1.**

**Additional file 2.**

**Additional file 3.**

**Additional file 4.**

**Additional file 5.**

**Additional file 6.**

**Additional file 7.**

**Additional file 8.**

**Additional file 9.**

**Additional file 10.**

**Additional file 11.**

**Additional file 12.**

**Additional file 13.**

**Additional file 14.**

**Additional file 15.**

**Additional file 16.**

**Additional file 17.**

**Additional file 18.**

## Data Availability

The datasets generated and analyzed during the current study are available in the NCBI repository, with the Accession Number SRP257840.

## Abbreviations

*ABA*: Abscisic acid
*ABC*: ATP Binding Cassette
*ADC*: Arginine decarboxylase
*AP2/ERF*: APETALA2/ethylene-responsive factor
*APX*: L-ascorbate peroxidase
*bHLH*: Basic helix-loop-helix
*BP*: Biological process
*BUSCO*: Benchmarking Universal Single-Copy Orthologs
*bZIP*: Basic leucine zipper
*CAT*: Catalase
*CC*: Cellular component
*COG*: Clusters of Orthologous Groups of proteins
*DAB*: Diaminobenzidine
*DEG*: Differentially expressed gene
*FC*: Fold change
*FW*: Formula weight
*GDS*: Germacrene D synthase
*GO*: Gene Ontology
*GPX*: glutathione peroxidase
*GRF*: growth-regulating factors
*GRX*: Glutaredoxin
*GST*: Glutathione S-transferase
*KEGG*: Kyoto Encyclopedia of Genes and Genomes
*MF*: Molecular function
*NBT*: Nitroblue tetrazolium
*NCBI*: National Center for Biotechnology Information
*NR*: NCBI non-redundant sequence
*NT*: NCBI nucleotide sequence
*P5CDH*: Pyrroline-5-carboxylate dehydrogenase
*P5CR*: Pyrroline-5-carboxylate reductase
*P5CS*: Pyrroline-5-carboxylate synthase
*PAO*: Primary-amine oxidase
*PEG*: Polyethylene glycol
*PEX*: Peroxisome
*Pfam*: The Protein families database
*POD*: Peroxidase
*ProDH*: Proline dehydrogenase
*PrxR*: Peroxiredoxin
*qRT-PCR*: Quantitative Real-time Polymerase Chain Reaction
*RNA-seq*: RNA sequencing
*ROS*: Reactive oxygen species
*RWC*: Relative water content
*SOD*: Superoxide dismutase
*SpeE*: *S*permidine synthase
*SRA*: Short Read Archive
*SULTR*: Sulfate transporter
*TF*: Transcription factor
*TP*: Transporter protein
*TPM*: Transcripts per million reads
*Trx*: Thioredoxin
*UGT*: UDP-glycosyltransferase
