# Supplementary material for: Iris lactea var. chinensis plant drought tolerance depends on the response of proline metabolism, transcription factors, transporters and the ROS-scavenging system
Source: BMC Plant Biol. 2023 Jan 9;23:17. doi: 10.1186/s12870-022-04019-4 (PMC9827652; doi:10.1186/s12870-022-04019-4)
Supplement: Supplementary file 1 — Additional file 1. [file 12870_2022_4019_MOESM1_ESM.docx]

**Table S1. Result of the *de novo* transcriptome assembly**

| Type | Resource |
| --- | --- |
| Total transcripts number | 180,769 |
| Total unigenes number | 126,979 |
| Total sequence base | 146,455,511 |
| Largest (bp) | 13,536 |
| Smallest (bp) | 201 |
| Average length (bp) | 810.18 |
| N50 (bp) | 1,176 |
| GC percent (%) | 42.55 |
| Mean mapped reads | 839.46 |
