# Supplementary figures and images for: Iris lactea var. chinensis plant drought tolerance depends on the response of proline metabolism, transcription factors, transporters and the ROS-scavenging system
Source: BMC Plant Biol. 2023 Jan 9;23:17. doi: 10.1186/s12870-022-04019-4 (PMC9827652; doi:10.1186/s12870-022-04019-4)

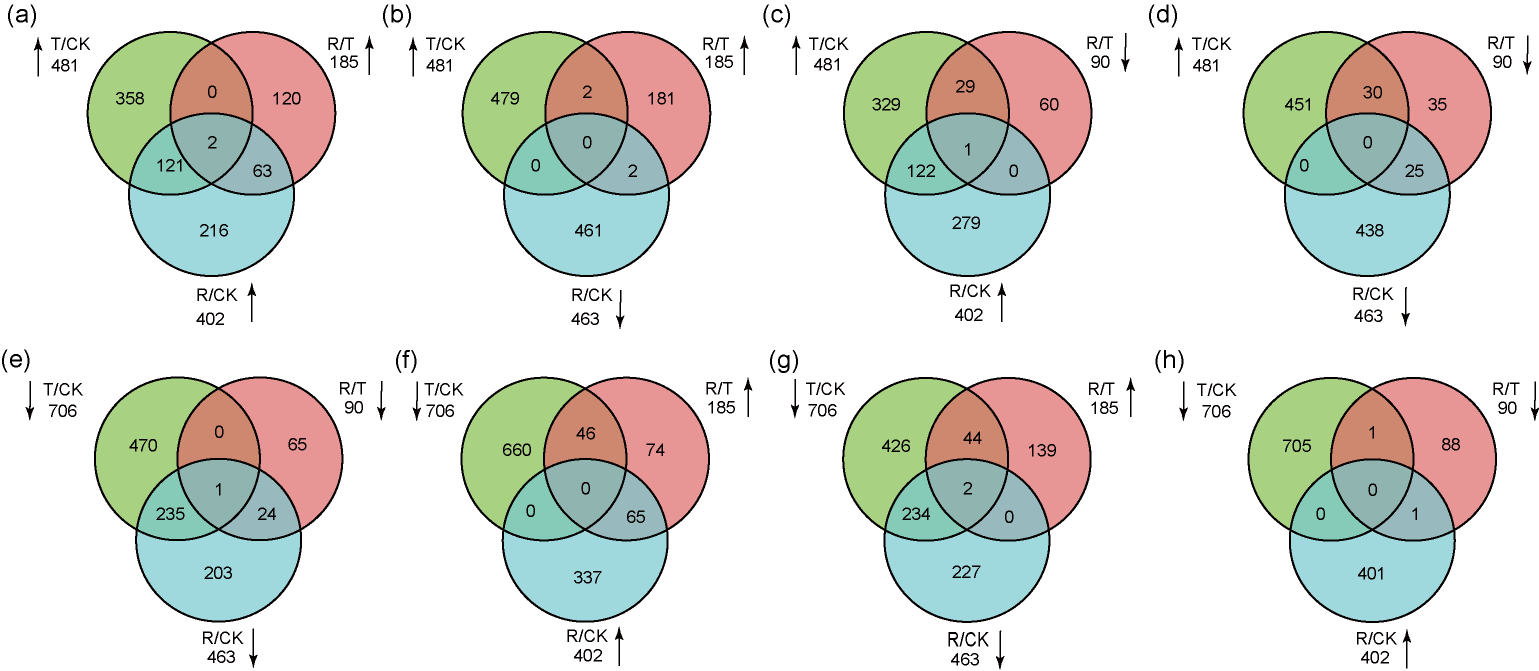

Supplement: Supplementary file 5 — Additional file 5. [file 12870_2022_4019_MOESM5_ESM.bmp]
