# Supplementary material for: Iris lactea var. chinensis plant drought tolerance depends on the response of proline metabolism, transcription factors, transporters and the ROS-scavenging system
Source: BMC Plant Biol. 2023 Jan 9;23:17. doi: 10.1186/s12870-022-04019-4 (PMC9827652; doi:10.1186/s12870-022-04019-4)
Supplement: Supplementary file 8 — Additional file 8. [file 12870_2022_4019_MOESM8_ESM.docx]

**Table S7. Enriched significantly GO terms in T (water-stressed) /CK (normal watering) (P-adjust<0.05)**

| GO ID | Term Type | Term | Number | P value | P-adjust |
| --- | --- | --- | --- | --- | --- |
| GO:0009065 | BP | glutamine family amino acid catabolic process | 6 | 5.438E-08 | 0.0003173^**^ |
| GO:0006562 | BP | proline catabolic process | 5 | 8.03E-07 | 0.0006104^**^ |
| GO:0008037 | BP | cell recognition | 17 | 8.03E-07 | 0.0006104^**^ |
| GO:0048544 | BP | recognition of pollen | 17 | 8.386E-07 | 0.0006104^**^ |
| GO:0022414 | BP | reproductive process | 21 | 8.386E-07 | 0.0006104^**^ |
| GO:0044702 | BP | single organism reproductive process | 20 | 1.035E-06 | 0.0006104^**^ |
| GO:0006259 | BP | DNA metabolic process | 16 | 1.102E-06 | 0.0006104^**^ |
| GO:0015074 | BP | DNA integration | 13 | 1.799E-06 | 0.0006104^**^ |
| GO:0044238 | BP | primary metabolic process | 114 | 2.072E-06 | 0.0006104^**^ |
| GO:0006807 | BP | nitrogen compound metabolic process | 74 | 2.257E-06 | 0.0006104^**^ |
| GO:0034641 | BP | cellular nitrogen compound metabolic process | 66 | 2.457E-06 | 0.0006104^**^ |
| GO:0043170 | BP | macromolecule metabolic process | 79 | 2.504E-06 | 0.0006104^**^ |
| GO:0044237 | BP | cellular metabolic process | 105 | 2.68E-06 | 0.0006104^**^ |
| GO:0044260 | BP | cellular macromolecule metabolic process | 74 | 2.735E-06 | 0.0006104^**^ |
| GO:0009987 | BP | cellular process | 151 | 2.738E-06 | 0.0006104^**^ |
| GO:0071704 | BP | organic substance metabolic process | 120 | 2.785E-06 | 0.0006104^**^ |
| GO:0006725 | BP | cellular aromatic compound metabolic process | 60 | 3.067E-06 | 0.0006104^**^ |
| GO:0006560 | BP | proline metabolic process | 5 | 3.147E-06 | 0.0006104^**^ |
| GO:1901566 | BP | organonitrogen compound biosynthetic process | 9 | 3.17E-06 | 0.0006104^**^ |
| GO:0006139 | BP | nucleobase-containing compound metabolic process | 59 | 3.294E-06 | 0.0006104^**^ |
| GO:0019538 | BP | protein metabolic process | 13 | 3.294E-06 | 0.0006104^**^ |
| GO:1901564 | BP | organonitrogen compound metabolic process | 18 | 3.296E-06 | 0.0006104^**^ |
| GO:0071472 | BP | cellular response to salt stress | 3 | 3.378E-06 | 0.0006104^**^ |
| GO:0046483 | BP | heterocycle metabolic process | 65 | 3.378E-06 | 0.0006104^**^ |
| GO:1901360 | BP | organic cyclic compound metabolic process | 67 | 3.471E-06 | 0.0006104^**^ |
| GO:0071470 | BP | cellular response to osmotic stress | 3 | 3.471E-06 | 0.0006104^**^ |
| GO:0071483 | BP | cellular response to blue light | 3 | 3.717E-06 | 0.0006104^**^ |
| GO:0044710 | BP | single-organism metabolic process | 39 | 3.777E-06 | 0.0006104^**^ |
| GO:0043604 | BP | amide biosynthetic process | 4 | 3.778E-06 | 0.0006104^**^ |
| GO:0010218 | BP | response to far red light | 3 | 3.932E-06 | 0.0006104^**^ |
| GO:0043603 | BP | cellular amide metabolic process | 5 | 3.995E-06 | 0.0006104^**^ |
| GO:0006412 | BP | translation | 4 | 4.031E-06 | 0.0006104^**^ |
| GO:0043043 | BP | peptide biosynthetic process | 4 | 4.031E-06 | 0.0006104^**^ |
| GO:0008152 | BP | metabolic process | 160 | 4.031E-06 | 0.0006104^**^ |
| GO:0009637 | BP | response to blue light | 4 | 4.076E-06 | 0.0006104^**^ |
| GO:0044267 | BP | cellular protein metabolic process | 12 | 4.092E-06 | 0.0006104^**^ |
| GO:0009639 | BP | response to red or far red light | 4 | 4.127E-06 | 0.0006104^**^ |
| GO:0006518 | BP | peptide metabolic process | 5 | 4.159E-06 | 0.0006104^**^ |
| GO:0090304 | BP | nucleic acid metabolic process | 57 | 4.16E-06 | 0.0006104^**^ |
| GO:0009553 | BP | embryo sac development | 3 | 4.185E-06 | 0.0006104^**^ |
| GO:0032774 | BP | RNA biosynthetic process | 41 | 4.618E-06 | 0.0006523^**^ |
| GO:0008150 | BP | biological_process | 243 | 4.779E-06 | 0.0006523^**^ |
| GO:0032991 | CC | macromolecular complex | 14 | 4.807E-06 | 0.0006523^**^ |
| GO:0044422 | CC | organelle part | 18 | 4.939E-06 | 0.0006550^**^ |
| GO:0044446 | CC | intracellular organelle part | 18 | 5.309E-06 | 0.0006713^**^ |
| GO:0016021 | CC | integral component of membrane | 220 | 5.363E-06 | 0.0006713^**^ |
| GO:0031224 | CC | intrinsic component of membrane | 220 | 5.407E-06 | 0.0006713^**^ |
| GO:0044425 | CC | membrane part | 222 | 5.624E-06 | 0.0006837^**^ |
| GO:0044444 | CC | cytoplasmic part | 37 | 6.613E-06 | 0.0007875^**^ |
| GO:0043234 | CC | protein complex | 9 | 7.212E-06 | 0.0008417^**^ |
| GO:0030529 | CC | intracellular ribonucleoprotein complex | 5 | 7.428E-06 | 0.0008498^**^ |
| GO:1990904 | CC | ribonucleoprotein complex | 5 | 8.791E-06 | 0.0009865^**^ |
| GO:0044424 | CC | intracellular part | 104 | 1.608E-05 | 0.0017255^**^ |
| GO:0044464 | CC | cell part | 123 | 1.626E-05 | 0.0017255^**^ |
| GO:0043228 | CC | non-membrane-bounded organelle | 5 | 1.626E-05 | 0.0017255^**^ |
| GO:0043232 | CC | intracellular non-membrane-bounded organelle | 5 | 1.68E-05 | 0.0017505^**^ |
| GO:0005840 | CC | ribosome | 3 | 2.251E-05 | 0.0023046^**^ |
| GO:0004657 | MF | proline dehydrogenase activity | 5 | 2.648E-05 | 0.0026636^**^ |
| GO:0004674 | MF | protein serine/threonine kinase activity | 45 | 3.013E-05 | 0.0029803^**^ |
| GO:0016773 | MF | phosphotransferase activity, alcohol group as acceptor | 87 | 3.207E-05 | 0.0031193^**^ |
| GO:0003723 | MF | RNA binding | 7 | 4.741E-05 | 0.0045350^**^ |
| GO:0004672 | MF | protein kinase activity | 83 | 5.506E-05 | 0.0051814^**^ |
| GO:0008270 | MF | zinc ion binding | 21 | 5.928E-05 | 0.0054905^**^ |
| GO:0016301 | MF | kinase activity | 93 | 7.898E-05 | 0.0070902^**^ |
| GO:0097367 | MF | carbohydrate derivative binding | 151 | 7.898E-05 | 0.0070902^**^ |
| GO:0032549 | MF | ribonucleoside binding | 143 | 0.0001187 | 0.0103343^*^ |
| GO:0001882 | MF | nucleoside binding | 143 | 0.0001187 | 0.0103343^*^ |
| GO:0005524 | MF | ATP binding | 134 | 0.0001276 | 0.0109475^*^ |
| GO:0032559 | MF | adenyl ribonucleotide binding | 136 | 0.0001409 | 0.0119115^*^ |
| GO:0030554 | MF | adenyl nucleotide binding | 136 | 0.0001504 | 0.0125400^*^ |
| GO:1901265 | MF | nucleoside phosphate binding | 156 | 0.0001556 | 0.012787^*^3 |
| GO:0000166 | MF | nucleotide binding | 156 | 0.0001605 | 0.0128264^*^ |
| GO:0032553 | MF | ribonucleotide binding | 149 | 0.0001863 | 0.0143834^*^ |
| GO:0003676 | MF | nucleic acid binding | 77 | 0.0001863 | 0.0143834^*^ |
| GO:0001883 | MF | purine nucleoside binding | 142 | 0.0001873 | 0.0143834^*^ |
| GO:0032555 | MF | purine ribonucleotide binding | 142 | 0.0001998 | 0.0151396^*^ |
| GO:0032550 | MF | purine ribonucleoside binding | 142 | 0.0002202 | 0.0164746^*^ |
| GO:0017076 | MF | purine nucleotide binding | 142 | 0.0002449 | 0.0180897^*^ |
| GO:0035639 | MF | purine ribonucleoside triphosphate binding | 140 | 0.0002725 | 0.0197382^*^ |
| GO:0036094 | MF | small molecule binding | 157 | 0.000274 | 0.0197382^*^ |
| GO:0043168 | MF | anion binding | 151 | 0.0003542 | 0.0252071^*^ |
| GO:0005198 | MF | structural molecule activity | 4 | 0.0003902 | 0.0274319^*^ |
| GO:0004714 | MF | transmembrane receptor protein tyrosine kinase activity | 5 | 0.0003988 | 0.0277030^*^ |
| GO:0016772 | MF | transferase activity, transferring phosphorus-containing groups | 101 | 0.0004107 | 0.0281959^*^ |
| GO:0016740 | MF | transferase activity | 162 | 0.0004626 | 0.0313878^*^ |
| GO:0004713 | MF | protein tyrosine kinase activity | 6 | 0.0006246 | 0.0418898^*^ |
| GO:0003735 | MF | structural constituent of ribosome | 4 | 0.0006488 | 0.0430229^*^ |
| GO:0016645 | MF | oxidoreductase activity, acting on the CH-NH group of donors | 6 | 0.0007181 | 0.0470779^*^ |
