# Supplementary material for: Iris lactea var. chinensis plant drought tolerance depends on the response of proline metabolism, transcription factors, transporters and the ROS-scavenging system
Source: BMC Plant Biol. 2023 Jan 9;23:17. doi: 10.1186/s12870-022-04019-4 (PMC9827652; doi:10.1186/s12870-022-04019-4)
Supplement: Supplementary file 9 — Additional file 9. [file 12870_2022_4019_MOESM9_ESM.docx]

**Table S8. Top 20 GO terms enriched in R (rehydration -treated) /T (water-stressed)**

| GO ID | Term Type | Term | P value | P-adjust |
| --- | --- | --- | --- | --- |
| GO:0016491 | MF | oxidoreductase activity | 3.074E-06 | 0.0025624^*^ |
| GO:0020037 | MF | heme binding | 2.681E-05 | 0.0130353^*^ |
| GO:0005576 | CC | extracellular region | 6.824E-05 | 0.0265443^*^ |
| GO:0046906 | MF | tetrapyrrole binding | 9.501E-05 | 0.0339055^*^ |
| GO:0008113 | MF | peptide-methionine (S)-S-oxide reductase activity | 0.0002426 | 0.0707842 |
| GO:0052837 | BP | thiazole biosynthetic process | 0.0003627 | 0.0785892 |
| GO:0052838 | BP | thiazole metabolic process | 0.0003627 | 0.0785892 |
| GO:0018131 | BP | oxazole or thiazole biosynthetic process | 0.0003627 | 0.0785892 |
| GO:0046484 | BP | oxazole or thiazole metabolic process | 0.0003627 | 0.0785892 |
| GO:0042744 | BP | hydrogen peroxide catabolic process | 0.0005395 | 0.104929 |
| GO:0042743 | BP | hydrogen peroxide metabolic process | 0.0005395 | 0.104929 |
| GO:0072593 | BP | reactive oxygen species metabolic process | 0.0009037 | 0.1633033 |
| GO:0009228 | BP | thiamine biosynthetic process | 0.0015651 | 0.2468255 |
| GO:0042724 | BP | thiamine-containing compound biosynthetic process | 0.0015651 | 0.2468255 |
| GO:0044712 | BP | single-organism catabolic process | 0.00182 | 0.2689476 |
| GO:0006772 | BP | thiamine metabolic process | 0.0018437 | 0.2689476 |
| GO:0042723 | BP | thiamine-containing compound metabolic process | 0.0018437 | 0.2689476 |
| GO:0005506 | MF | iron ion binding | 0.0019235 | 0.2737539 |
| GO:0004497 | MF | monooxygenase activity | 0.0023829 | 0.3310517 |
| GO:0016682 | MF | oxidoreductase activity, acting on diphenols and related substances as donors, oxygen as acceptor | 0.0033384 | 0.4427208 |
