# Supplementary material for: Iris lactea var. chinensis plant drought tolerance depends on the response of proline metabolism, transcription factors, transporters and the ROS-scavenging system
Source: BMC Plant Biol. 2023 Jan 9;23:17. doi: 10.1186/s12870-022-04019-4 (PMC9827652; doi:10.1186/s12870-022-04019-4)
Supplement: Supplementary file 10 — Additional file 10. [file 12870_2022_4019_MOESM10_ESM.docx]

**Table S9. Top 20 GO terms enriched in R (rehydration -treated) /CK (normal watering)**

| GO ID | Term Type | Term | P value | P-adjust |
| --- | --- | --- | --- | --- |
| GO:0035556 | BP | intracellular signal transduction | 7.56299E-06 | 0.011032505^*^ |
| GO:0000160 | BP | phosphorelay signal transduction system | 2.63673E-05 | 0.022744759^*^ |
| GO:0052837 | BP | thiazole biosynthetic process | 6.26749E-05 | 0.022856754^*^ |
| GO:0052838 | BP | thiazole metabolic process | 6.26749E-05 | 0.022856754^*^ |
| GO:0018131 | BP | oxazole or thiazole biosynthetic process | 6.26749E-05 | 0.022856754^*^ |
| GO:0046484 | BP | oxazole or thiazole metabolic process | 6.26749E-05 | 0.022856754^*^ |
| GO:0004672 | MF | protein kinase activity | 3.19685E-05 | 0.022856754^*^ |
| GO:0004674 | MF | protein serine/threonine kinase activity | 5.98853E-05 | 0.022856754^*^ |
| GO:0016021 | CC | integral component of membrane | 0.000175997 | 0.055358089 |
| GO:0031224 | CC | intrinsic component of membrane | 0.000180258 | 0.055358089 |
| GO:0016491 | MF | oxidoreductase activity | 0.000214798 | 0.062667317 |
| GO:0016773 | MF | phosphotransferase activity, alcohol group as acceptor | 0.000314696 | 0.079836898 |
| GO:0009228 | BP | thiamine biosynthetic process | 0.000645148 | 0.139423564 |
| GO:0042724 | BP | thiamine-containing compound biosynthetic process | 0.000645148 | 0.139423564 |
| GO:0006772 | BP | thiamine metabolic process | 0.000829488 | 0.158349831 |
| GO:0042723 | BP | thiamine-containing compound metabolic process | 0.000829488 | 0.158349831 |
| GO:0005739 | CC | mitochondrion | 0.000782556 | 0.158349831 |
| GO:0010181 | MF | FMN binding | 0.000928714 | 0.169345272 |
| GO:0009846 | BP | pollen germination | 0.001291025 | 0.224564344 |
| GO:0044425 | CC | membrane part | 0.001439511 | 0.239987108 |
