# Supplementary material for: Iris lactea var. chinensis plant drought tolerance depends on the response of proline metabolism, transcription factors, transporters and the ROS-scavenging system
Source: BMC Plant Biol. 2023 Jan 9;23:17. doi: 10.1186/s12870-022-04019-4 (PMC9827652; doi:10.1186/s12870-022-04019-4)
Supplement: Supplementary file 11 — Additional file 11. [file 12870_2022_4019_MOESM11_ESM.docx]

**Table S10. Statistical enrichment analysis for KEGG pathways in T (water-stressed) /CK (normal watering)**

| Number | iD | Term | P value | P -adjust |
| --- | --- | --- | --- | --- |
| 20 | ko04626 | Plant-pathogen interaction | 1.198E-06 | 0.0000839^**^ |
| 9 | ko00592 | alpha-Linolenic acid metabolism | 1.137E-05 | 0.00039^**^ |
| 8 | ko04712 | Circadian rhythm - plant | 0.0005317 | 0.0124^*^ |
| 12 | ko02010 | ABC transporters | 0.0012263 | 0.02146^*^ |
| 7 | ko00330 | Arginine and proline metabolism | 0.003822 | 0.04459^*^ |
| 8 | ko00480 | Glutathione metabolism | 0.0035664 | 0.04993^*^ |
| 14 | ko04075 | Plant hormone signal transduction | 0.0079252 | 0.07925 |
| 8 | ko04146 | Peroxisome | 0.0138732 | 0.12139 |
| 9 | ko04016 | MAPK signaling pathway - plant | 0.0304286 | 0.23666 |
| 14 | ko04141 | Protein processing in endoplasmic reticulum | 0.0378511 | 0.26495 |
