# Supplementary material for: Iris lactea var. chinensis plant drought tolerance depends on the response of proline metabolism, transcription factors, transporters and the ROS-scavenging system
Source: BMC Plant Biol. 2023 Jan 9;23:17. doi: 10.1186/s12870-022-04019-4 (PMC9827652; doi:10.1186/s12870-022-04019-4)
Supplement: Supplementary file 12 — Additional file 12. [file 12870_2022_4019_MOESM12_ESM.docx]

**Table S11. Statistical enrichment analysis for KEGG pathways in R (rehydration -treated) /T (water-stressed)**

| Number | iD | Term | P value | P -adjust |
| --- | --- | --- | --- | --- |
| 8 | ko00940 | Phenylpropanoid biosynthesis | 1.138E-05 | 0.0004097^**^ |
| 3 | ko00960 | Tropane, piperidine and pyridine alkaloid biosynthesis | 0.0004872 | 0.0087701^**^ |
| 8 | ko04141 | Protein processing in endoplasmic reticulum | 0.0026803 | 0.0241229^*^ |
| 3 | ko00941 | Flavonoid biosynthesis | 0.0025597 | 0.0307164^*^ |
| 2 | ko00730 | Thiamine metabolism | 0.0192718 | 0.0991119 |
| 2 | ko00945 | Stilbenoid, diarylheptanoid and gingerol biosynthesis | 0.0170132 | 0.1020789 |
| 3 | ko00260 | Glycine, serine and threonine metabolism | 0.0249183 | 0.1121323 |
| 2 | ko00950 | Isoquinoline alkaloid biosynthesis | 0.0155751 | 0.1121407 |
| 2 | ko00591 | Linoleic acid metabolism | 0.0303849 | 0.1215395 |
| 2 | ko00100 | Steroid biosynthesis | 0.0456147 | 0.1642128 |
