# Supplementary material for: Iris lactea var. chinensis plant drought tolerance depends on the response of proline metabolism, transcription factors, transporters and the ROS-scavenging system
Source: BMC Plant Biol. 2023 Jan 9;23:17. doi: 10.1186/s12870-022-04019-4 (PMC9827652; doi:10.1186/s12870-022-04019-4)
Supplement: Supplementary file 13 — Additional file 13. [file 12870_2022_4019_MOESM13_ESM.docx]

**Table S12. Statistical enrichment analysis for KEGG pathways in R (rehydration -treated) /CK (normal watering)**

| Number | iD | Term | P value | P -adjust |
| --- | --- | --- | --- | --- |
| 13 | ko04712 | Circadian rhythm - plant | 2.634E-09 | 1.738E-07^**^ |
| 20 | ko04141 | Protein processing in endoplasmic reticulum | 1.011E-05 | 0.0003335^**^ |
| 13 | ko03040 | Spliceosome | 0.0022327 | 0.0491193^*^ |
| 7 | ko00250 | Alanine, aspartate and glutamate metabolism | 0.003283 | 0.0541691 |
| 11 | ko04626 | Plant-pathogen interaction | 0.0058812 | 0.0646931 |
| 5 | ko00592 | alpha-Linolenic acid metabolism | 0.0052949 | 0.0698926 |
| 8 | ko00940 | Phenylpropanoid biosynthesis | 0.0092926 | 0.0876157 |
| 11 | ko04144 | Endocytosis | 0.015012 | 0.123849 |
| 3 | ko00945 | Stilbenoid, diarylheptanoid and gingerol biosynthesis | 0.0172445 | 0.1264593 |
| 3 | ko00730 | Thiamine metabolism | 0.0205501 | 0.1356309 |
